# Volatile organic compounds from *Streptomyces rochei* TRM68247: fumigant biocontrol against postharvest diseases of Korla fragrant pear and identification of active antifungal components

**DOI:** 10.3389/fmicb.2026.1930527

**Published:** 2026-10-06

**Authors:** Yi Huang Chen, Chao Wen Liu, Jin Duo Zhao, Tian Cai Wang, Yu Xin Zhang, Wei Huang, XiaoXia Luo

**Affiliations:** School of Life Sciences and Technology, Tarim University, Alar, China

**Keywords:** biocontrol, pear rot disease, *Streptomyces rochei*, volatile organic compounds (VOCs), whole-genome analysis

## Abstract

**Background:**

Long-term application of synthetic fungicides leads to severe residue risks and pathogen drug resistance during postharvest storage of Korla fragrant pear (*Pyrus sinkiangensis*), making it urgent to develop low-residue gaseous biopreservation agents. This study investigated the fumigant biocontrol capacity of volatile organic compounds (VOCs) produced by desert-derived *Streptomyces rochei* TRM68247 against two dominant postharvest pathogens, *Valsa pyri* and *Alternaria gaisen*.

**Methods:**

Whole-genome sequencing was performed to identify biosynthetic gene clusters (BGCs) associated with volatile secondary metabolism. Volatile compounds were profiled by GC-MS, and core bioactive substances were screened and validated by *in vitro* dual-plate assays. Fumigation efficacy was further evaluated on pear twigs and on wounded, inoculated fruits under simulated storage conditions, with commercial eugenol included as a positive control.

**Results:**

Strain TRM68247 harboured abundant BGCs supporting stable production of antifungal volatiles under storage conditions. GC-MS identified 44 volatile components, among which 2-ethyl-1-hexanol and 3,5-di-tert-butylphenol were screened as core bioactive substances. *In vitro* dual-plate tests showed that 2-ethyl-1-hexanol exerted broad and strong inhibitory activity against both fungi. Fumigation with VOCs from TRM68247 significantly retarded lesion expansion and blocked deep pulp infection, and the control efficacy against fruit black spot was comparable to that of commercial eugenol.

**Conclusions:**

VOCs from *S. rochei* TRM68247 are promising candidate fumigants for reducing postharvest rot loss of Korla fragrant pear, and the present work supplies two natural volatile antifungal compounds for the development of green fruit-preservation technology.

## Graphical Abstract

**Figure:**
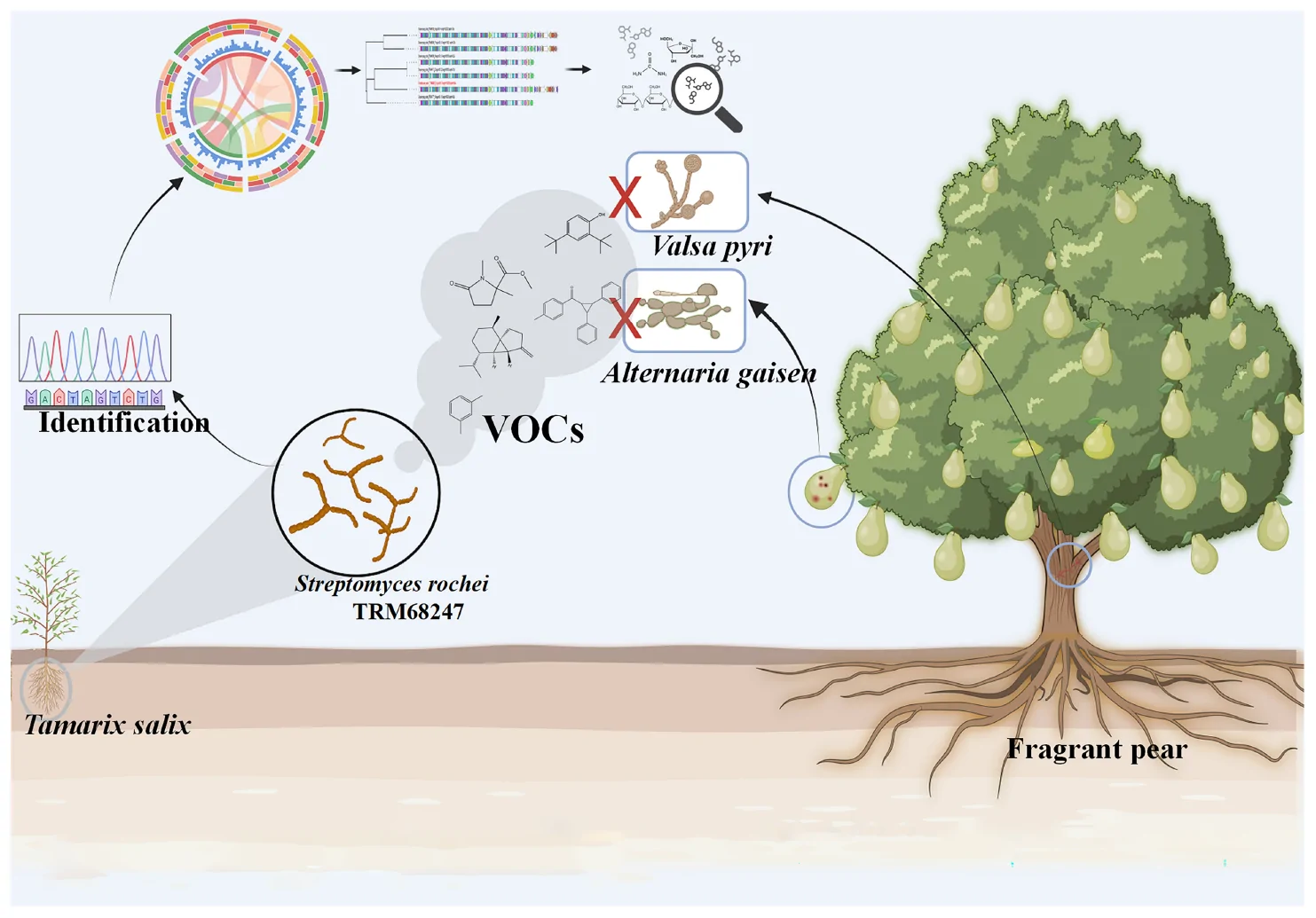
The VOC s Produced by *Streptomyces rochei* TRM68247 can inhibit *Valsa pyri* and *Alternaria gaisen*, thus showing potential as a new pesticide.

## 1 Introduction

Fragrant pear is a key agricultural commodity in Xinjiang (China) and occupies an important position in the local fruit storage and processing industry (Jiang et al., 2023). Postharvest rot and black spot diseases, mainly caused by *Valsa pyri* and *Alternaria gaisen*, frequently occur during pear fruit storage and transportation. These two pathogens severely damage fruit quality, shorten shelf life and lead to huge economic losses for the fragrant pear industry (He et al., 2022). Carbendazim and tebuconazole are commonly sprayed to control pear diseases during field cultivation. After harvest, chemical preservatives including prochloraz, imazalil, and pyrimethanil are widely used via fruit dipping or warehouse fumigation to suppress postharvest rot and black spot of fragrant pear (Yuan et al., 2021, 2022). Nevertheless, prolonged and excessive application of chemical agents has induced widespread drug resistance in pathogens. Continuous reliance on single fungicides has further aggravated the resistance of *V. pyri* and *A. gaisen* (Shi et al., 2024). To maintain control efficacy, farmers have to increase dosage or use more potent pesticides, which raises the overall storage cost. In addition, chemical pesticides inevitably cause residual problems and environmental pollution, threatening ecological safety and human health. Therefore, exploring safe, efficient and sustainable biopreservation technologies has become an urgent demand for the fragrant pear industry. Biocontrol fumigation can reduce chemical dependence and mitigate environmental risks, which conforms to the green development concept of fruit postharvest storage.

*S. rochei* has attracted extensive attention in agricultural biocontrol. Its genome harbors abundant biosynthetic gene clusters for secondary metabolites, conferring strong antifungal and plant growth-promoting capacities (Shi et al., 2024). Previous studies proved that *S. rochei* could synthesize diverse natural products and suppress multiple soil-borne crop diseases such as cucumber fusarium wilt and pepper phytophthora root rot via fermentation filtrate or single purified metabolites (Bubici, 2018; Zhang et al., 2024; Zhu et al., 2024; Shirokikh et al., 2023). Various bioactive compounds including borrelidin and streptothricin have been characterized from different *S. rochei* strains, while traditional extraction methods limit the discovery of novel metabolites (Singh et al., 1983; Han et al., 2021; Zhang et al., 2025; Liu et al., 2012; Anukool et al., 2004). All these findings verify that *S. rochei* is a promising microbial resource. However, existing research on this species mainly focuses on field disease control, and few studies have explored its volatile organic compounds (VOCs) for fruit postharvest fumigation preservation.

Microbial VOCs are considered promising candidates for developing eco-friendly biological fumigants in postharvest disease management (Gomiero et al., 2011). As gaseous compounds, microbial VOCs can exert antifungal activity via non-contact diffusion. Previous literature suggested the potential advantages of microbial volatile-based fumigants for fruit storage. Relevant studies have provided theoretical support for this field: volatiles from *Streptomyces albulus* Z1-04-02 achieved high inhibition efficiency against *Sclerotinia sclerotiorum* by inducing cell wall-degrading enzyme secretion (Ruangwong et al., 2022). VOCs produced by rhizospheric *S. rochei* ASH destroyed the hyphal structure of *Fusarium graminearum* and reduced the disease severity index significantly (Sudha et al., 2022). Nevertheless, few studies have focused on VOCs from desert-derived actinomycetes for concurrent suppression of the two key postharvest pathogens of Korla fragrant pear.

Currently, research on VOC-mediated control of fragrant pear postharvest rot and black spot remains scarce. Most reported antagonistic microbes are isolated from conventional agricultural soils, and may exhibit limited environmental adaptability under fluctuating temperature and humidity encountered during fruit storage. To date, only volatiles from *Aspergillus niger* La2 have been documented to inhibit *V. pyri*, whereas the co-infection scenario caused by *A. gaisen* was not considered in that study (Shi et al., 2024). Most existing studies focus primarily on chemical fungicides or liquid microbial agents, with few systematic evaluations of VOC fumigation efficacy and antifungal mechanisms targeting pear postharvest pathogens. To address this research gap, we screened the indigenous desert strain *S. rochei* TRM68247 isolated from *Tamarix ramosissima* rhizosphere soil, which displays strong antagonistic activity against both *V. pyri* and *A. gaisen*. In the present study, we performed strain identification, whole-genome functional analysis, and bioassays for its antagonistic volatile metabolites. This work aims to provide a theoretical basis and technical reference for exploring candidate gaseous fumigants and advancing the green postharvest preservation of fragrant pear.

## 2 Materials and methods

### 2.1 Isolation and preservation

*S. rochei* TRM68247 was isolated from the rhizosphere soil of *T. ramosissima* and cultured on International *Streptomyces* Project 4 (ISP4) plates (containing 10 g/L soluble starch, 1 g/L K_2_HPO_4_, 1 g/L MgSO_4_, 1 g/L NaCl, 2 g/L (NH4)_2_SO_4_, 2 g/L CaCO_3_, 1 mL/L trace salt solution, and 18 g/L agar). Its genome has been deposited under the accession number JBNGCE000000000. *V. pyri* TRM55784 and *A. gaisen* TRM58866 were originally isolated from diseased pear (*Pyrus sp.*) tissues collected in the pear orchard of Tarim University, Alar, Xinjiang, China (approximately 40.55°N, 81.30°E). Potato Dextrose Agar (PDA) plates (containing 200 g/L potato, 20 g/L glucose, 15–20 g/L agar) were used for activation and cultivation of these two pathogenic strains. All strains, including S. rochei TRM68247, *V. pyri*, and *A. gaisen*, are preserved in the State Key Laboratory of Tarim University for the Conservation and Utilization of Biological Resources in the Tarim Basin.

### 2.2 Identification of strain TRM68247

Morphological identification: Strain TRM68247 was cultured on Gauze’s Synthetic Medium No. 1 (containing 20 g/L soluble starch, 1 g/L KNO_3_, 0.5 g/L K_2_HPO_4_, 0.5 g/L MgSO_4_⋅7H_2_O, 0.5 g/L NaCl, 0.01 g/L FeSO_4_⋅7H_2_O, 18 g/L agar; pH 7.2–7.4) at 30 °C for 4 days, after which the colony morphological characteristics were observed and recorded. Bacterial blocks with intact colonies (0.5 cm × 0.5 cm) were cut with a sterile blade and immediately placed into a PA bottle. Pre-cooled 2.5% glutaraldehyde fixative was added at 4 °C to fully immerse the samples, followed by overnight fixation at 4 °C (12–16 h). After fixation, the samples were dehydrated sequentially in 30%, 50%, 70%, 80%, 90%, and 95% ethanol solutions for 15 min each and finally twice in absolute ethanol for 15 min each time. The dehydrated samples were then subjected to critical point drying to remove residual moisture, gold-sputtered using an ion sputtering coater, and observed under scanning electron microscopy (SEM, Thermo Fisher Scientific, Waltham, MA, USA) at an accelerating voltage of 10–15 kV to characterize the morphological features of hyphae and spores.

Molecular biological identification: Genomic DNA of TRM68247 was extracted using the CTAB method (Prins et al., 2024). PCR amplification of the 16S rDNA gene was conducted with universal primers and the extracted DNA as the template (Rintala et al., 2001). The resulting products were validated by agarose gel electrophoresis before being submitted to Shanghai Sangon Biotech Co., Ltd. for sequencing. The obtained 16S rDNA sequence was compared against the EzBioCloud database^1^, which returned 19 closely related sequences. A phylogenetic tree was then generated using the Maximum Likelihood (ML) method in MEGA X software (version 10.2) (Ranneby, 1984).

### 2.3 Genomic genetic characteristics analysis of strain TRM68247

Strain TRM68247 was cultured in ISP4 liquid medium (pH 7.2; the liquid and solid ISP4 media had identical compositions except that agar was added to the solid medium) at 30 °C with shaking at 200 r/min for 6 days. Mycelial pellets were collected by centrifugation at 8,000 r/min for 10 min, and whole-genome sequencing was then performed by Nanjing Personal Biotechnology Co., Ltd. (Nanjing, China). Sequencing was conducted on the Illumina HiSeq 2000 platform, and genome assembly, gap filling, and functional annotation were performed with SPAdes v3.15.2, GapCloser v1.12, and Prokka v1.14.6, respectively.

The whole-genome reference sequences of the 18 most closely related strains to TRM68247 were downloaded from NCBI^2^ and uploaded to the IPGA v1.09 online platform (Liu et al., 2022) of the National Microbiology Data Center on 2 March 2025. Quality control was implemented to ensure a genome completeness of ≥90% and contamination of ≤5%. Comparative genomics analysis was performed using built-in tools such as PanOCT and OrthFinder with default parameters, and genomic indices, including average nucleotide identity (ANI), were computed. A whole-genome phylogenetic tree was constructed based on core genes, followed by pan-genome analysis. The tree was visualized using iTOL v6.7, and pan-genome fitting curves were generated in Origin 2024. Pan-genome openness was assessed by a power-law regression function obtained from the Levenberg–Marquardt optimization algorithm. Insertion sequences within the TRM68247 genome were identified and subjected to evolutionary adaptability analysis using the gcPathogen online platform (Guo et al., 2024).

### 2.4 Metabolic potential analysis of strain TRM68247

The online antiSMASH database^3^ was used to predict biosynthetic gene clusters (BGCs) associated with secondary metabolite production in strain TRM68247, and the results were statistically analyzed to deduce potential metabolites (Blin et al., 2021). Antibiotic resistance genes in TRM68247 were identified using the Comprehensive Antibiotic Resistance Database (CARD^4^) (Alcock et al., 2020), and subsequent functional annotation and distribution analysis were conducted to assess the strain’s antibiotic resistance capabilities.

### 2.5 Activity detection of TRM68247 volatiles against *V. pyri*

The double-plate confrontation assay was used to evaluate the fumigant activity of volatile compounds produced by strain TRM68247 (Nazik et al., 2020). Briefly, strain TRM68247 was inoculated onto Gauze’s Synthetic Medium No. 1 plates and incubated at 28 °C for 4 days. Spores were harvested and suspended in sterile water, and the spore suspension was adjusted to 0.5 McFarland units using a turbidimeter. A 100-μL aliquot of the spore suspension was spread evenly onto an ISP4 plate, which served as the lower plate. A PDA plate (upper plate) was centrally inoculated with a 7-mm-diameter mycelial plug of *V. pyri* or *A. gaisen*. The two plates were then placed mouth-to-mouth with the upper plate inverted, so that the two media surfaces faced each other without physical contact, and were sealed along the rim Parafilm Parafilm to form a closed, shared-headspace system. This airtight sealing, together with the absence of direct contact between the two media surfaces, prevented any diffusible or airborne transfer of cells between the two compartments, so that the test pathogens were exposed only to the gas phase of the system. The actual gaseous exposure of the pathogens is affected by the sealed headspace volume, compound volatility, partitioning behavior, temperature, adsorption to the medium/plate surfaces, and incubation duration. For the blank control (CK), the lower plate contained plain ISP4 medium without TRM68247 spores, while all other operations remained identical. The paired plates were kept at 28 °C, and the growth of *V. pyri* and *A. gaisen* was monitored and recorded at 2, 4, and 8 days. The growth inhibition rate of the pathogens was calculated using Equation 1 relative to the control, and the inhibitory effect associated with the TRM68247 paired-culture system was thereby evaluated. It should be noted that, although the sealed non-contact configuration excluded direct microbial interaction, and although the headspace VOC profiles (section “2.7 Collection and analysis of VOCs from strain TRM68247”) and the fumigant activity of authentic compounds (section “2.8 Verification of the activity of potential VOCs”) are consistent with a volatile-mediated mechanism, the paired-plate assay by itself cannot identify which volatile component(s) caused the inhibition. Accordingly, the Results refer to inhibition associated with the TRM68247 paired-culture system


$$\text{I}\mathrm{nhibition}\mathrm{rate}\left(\%\right)=\left(1-\frac{\mathrm{Colony}\,\mathrm{diameter}\,\text{intreatment}}{\mathrm{Colony}\,\mathrm{diameter}\,\text{incontrol}}\right)\times 100$$ (1)

### 2.6 Evaluation of biocontrol efficacy sealed-culture system against detached pear tissue samples

#### 2.6.1 Biocontrol efficacy against valsa canker on young ear shoots

The preserved *V. pyri* strain was inoculated onto PDA plates and cultured in a constant-temperature incubator at 28 °C for 4 days for activation. After the colonies grew sufficiently, uniform mycelial plugs were cut along the edge of the colonies with a sterile punch [diameter (d) = 7 mm] and set aside. Strain TRM68247 was inoculated onto Gauze’s Synthetic Medium No. 1 plates [diameter (d) = 9 mm] and cultured at 30 °C for 4 days for subsequent biocontrol detection; meanwhile, blank Gauze’s Synthetic Medium No. 1 plates without the strain were prepared as the carrier for the control experiment.

Healthy young pear shoots with uniform growth were collected from the Horticultural Experiment Station of Tarim University. After being rinsed thoroughly with running water, the shoots were cut into 15-cm-long segments and placed in an ultra-clean workbench. The surface of the segments was disinfected by wiping with 75% ethanol, rinsed three times with sterile water, and blotted dry with sterile filter paper; the two ends of the segments were sealed with melted paraffin to prevent water loss and bacterial contamination. A sterile punch [diameter (d) = 7 mm] was used to prepare one wound at the central position of each shoot.

A closed desiccator fumigation assay, adapted from the method of Huang et al. (2022) with slight modifications, was used for pathogen-inoculated pear shoots. A desiccator (inner diameter 240 mm; approximate internal volume 3.8 L) was sterilized by wiping the inner wall with 75% ethanol and air-dried naturally. For the treatment group, five agar plates were placed on the bottom of the desiccator, each containing 10 mL of Gauze’s Synthetic Medium No. 1 and inoculated with 1 mL of TRM68247 fermentation broth standardized to 1 × 108 CFU/mL. For inoculation, the wounds on the young pear shoots were tightly covered with *V. pyri* mycelial plugs with the mycelial side facing downward, and the inoculated shoots were then placed inside the desiccator without contacting the agar plates or the inner wall; the strain culture and the plant tissue were therefore separated solely by the shared headspace. The desiccator lid was closed rapidly, and the junction was sealed with vaseline (Yucai Pharmaceutical Co., Ltd., China) to form an airtight chamber, excluding any direct contact or transfer of culture material to the shoots.

Three treatment groups were established, with three biological replicates per group (10 shoots per replicate). For the negative control group (CK1), blank Gauze’s Synthetic Medium No. 1 plates replaced the strain-inoculated plates, while all other operations remained identical. For the positive control group, 1 mL of eugenol (purity ≥ 98%, Coolaber Science & Technology, Shanghai, China) was placed at the bottom of the desiccator, with all other experimental procedures kept consistent. Eugenol was used only as a positive reference, and a strict dose-equivalent comparison of volatile potency between pure eugenol and strain-released VOCs was not performed. All sealed desiccators were incubated in darkness at a constant temperature of 28 °C. Disease development on pear shoots was recorded at day 7 and day 14 post-incubation, and lesion length (mm) at the inoculated wounds was measured using the cross-measurement method.

#### 2.6.2 Biocontrol efficacy against *A. gaisen* on pear fruits

Fragrant pear fruits were purchased from the market, and healthy individuals with uniform maturity and size, a single fruit weight of 200 ± 30 g, and no mechanical damage were selected. The fruits were rinsed with clean water to remove surface dirt, surface-sterilized with 75% ethanol, rinsed three times with sterile water, and air-dried naturally in an ultra-clean workbench for later use. A wound (3 mm in depth and 3 mm in diameter) was made at the equator of each fruit with a sterile inoculating needle.

The *A. gaisen* strain was spread on PDA medium and cultured at 28 °C for 4 days. Spores and mycelia on the colony surface were scraped off, and a spore–mycelium mixture was prepared with sterile water. The concentration of the mixture was adjusted to 0.5 McFarland units by the McFarland turbidity method, and the suspension was set aside. Mature colony plates of strain TRM68247 were prepared as described in section “2.6.1 Biocontrol efficacy against valsa canker on young ear shoots” and set aside. The pretreated pear fruits were placed on a sterile tray in the desiccator so that the fruits did not come into contact with the medium or the inner wall of the desiccator. A 10-μL aliquot of the prepared *A. gaisen* spore–mycelium suspension was added dropwise to the wound of each fruit and spread evenly with a sterile cotton swab to ensure that the suspension completely covered the wound. The desiccator was quickly covered, and the joint was sealed with vaseline. The sealed desiccators were incubated at 28 °C to accelerate disease development. Three treatment groups were established, with three biological replicates in each group (three fruits per replicate). The inhibition rate was calculated using Equation 2. Lesion area, the proportion or volume of rotten tissue, and a predefined ordinal disease-severity index were not quantified in the present study; these measures, together with blinded assessment, are acknowledged as limitations and will be addressed in future work.


Inhibitionrate(%)= (2)

$$\frac{\mathrm{Lesion}\,\mathrm{diameter}\,\mathrm{of}\,\mathrm{negative}\,\mathrm{control}-\mathrm{Lesion}\,\mathrm{diameter}\,\mathrm{of}\,\mathrm{treatment}}{\mathrm{Lesion}\,\mathrm{diameter}\,\mathrm{of}\,\mathrm{negative}\,\mathrm{control}}\times 100$$

### 2.7 Collection and analysis of VOCs from strain TRM68247

Strain TRM68247 was cultured in ISP4 liquid medium at 30 °C and 150 r/min for 4 days. Then, 100 μL of the fermentation broth was inoculated into a 20-mL sterile headspace vial containing Gauze’s Synthetic Medium No. 1 and incubated at 30 °C for another 4 days. Meanwhile, blank controls containing only Gauze’s Synthetic Medium No. 1 were incubated under identical conditions. Volatile organic compounds (VOCs) were extracted via solid-phase microextraction (SPME) using an AOC-600Plus SPME autosampler (Shimadzu, Japan) with a Smart SPME fiber (80 μm DVB/C-WR/PDMS coating). Before use, the SPME fiber was conditioned according to the manufacturer’s recommendation to remove residual contaminants. Pre-equilibration was performed at 75 °C for 5 min, followed by adsorption extraction for 30 min. After extraction, the fiber was transferred to the GC inlet for desorption at 250 °C for 5 min. GC-MS analysis was performed on a Shimadzu GCMS-QP2020NX instrument (Shimadzu, Japan) equipped with a DB-5MS capillary column (30 m × 0.25 mm i.d., 0.25 μm film thickness). High- purity helium was used as the carrier gas at a constant flow rate of 1 mL min^(−1^. The oven temperature was held at 40 °C for 3 min, increased to 280 °C at 10 °C min^(−1^, and held at 280 °C for 10 min. Splitless injection mode was adopted, and an electron ionization (EI) ion source was used for mass detection. Volatile components were identified by matching mass spectra against the NIST26 Mass Spectral Library (NIST Standard Reference Database 1A, 2026 version). Notably, the relative proportions of individual VOCs were determined via peak area normalization. This method reflects relative compositional abundance rather than absolute production quantities. Owing to compound-specific differences in SPME adsorption affinity, volatility, and EI-MS detector response factors, the obtained relative ratios may not fully represent the actual *in vivo* emission amounts of each volatile component. Candidate antimicrobial substances were screened based on their chemical structures and previously reported biological activities.

### 2.8 Verification of the activity of potential VOCs

Each candidate compound (nonanal, 2,3-dihydro-1H-inden-1-ol, 2-ethyl-1-hexanol, 3,5-di-tert-butylphenol, and isomenthene) was purchased from Shanghai Yuanye Bio-Technology Co., Ltd. (Shanghai, China), with purity >90%. Each compound (0.05 g) was weighed into a 2-mL sterile centrifuge tube and dissolved in 1 mL of chromatographically pure methanol with vortexing for 10 min to yield a stock solution of 50 mg/mL (5 × 104 μg/mL). Five tenfold serial dilutions were then prepared in methanol, giving working concentrations of 5 × 10^2^, 5 × 10^−1^, 5, 5 × 10^(−1^, and 5 × 10^(2^ μg/mL. The dual-compartment plate assay was used to assess the fumigant inhibitory activity of the target compounds against *V. pyri* and *A. gaisen* (Kavanagh, 1947). Briefly, a disposable dual-compartment culture plate was used. Sterilized PDA medium was poured into one compartment; after solidification, an activated pathogenic mycelial plug (diameter = 7 mm) was inoculated onto the medium. A sterilized square filter paper (15 mm × 15 mm) was placed near the far edge of the second compartment, and a 200-μL aliquot of each working solution was pipetted and evenly loaded onto the filter paper (corresponding to applied masses of 100, 10, 1, 0.1, and 0.01 μg per chamber at the five concentrations, respectively) to ensure full absorption without liquid overflow. After sample loading, the plate junction was tightly sealed with PVC wrapping film to establish a closed headspace system. A blank control was set up in which an equal volume of chromatographically pure methanol without the target compound was applied to the filter paper; all other experimental operations were identical to those of the treatment groups. All plates were incubated statically in a constant-temperature incubator at 28 °C. Once colonies in the blank control grew stably, the colony areas of the pathogenic fungi were quantified using ImageJ software^5^, with the partition boundary of the dual-compartment plate (85.00 mm) as the reference. Each treatment was represented by three independent dual-compartment plates (biological replicates, *n* = 3), and the colony area of each plate was quantified once by ImageJ; repeated pixel measurements of the same plate were not treated as biological replicates. The inhibition rate was calculated according to Equation 3, and the volatile minimum inhibitory concentration (VMIC) of the target compounds was determined. The exact internal headspace volume of the sealed dual-compartment plate was not precisely determined, because the free gas space was partially occupied by the solidified PDA medium and the filter paper. Accordingly, the doses reported here refer to the methanolic working solutions loaded onto the filter paper and the corresponding absolute applied mass per chamber (100, 10, 1, 0.1, and 0.01 μg), rather than to the actual gaseous concentration of the compound in the closed headspace. The real gaseous exposure of the pathogens is affected by the applied compound mass, sealed headspace volume, compound volatility, partitioning behavior, temperature, adsorption to the filter paper, medium and plate surfaces, and incubation duration.

Volatile minimum inhibitory concentration is defined as the lowest concentration of the methanolic working solution of volatile compounds that significantly inhibits mycelial growth of the pathogens and limits colony area to ≤5% of the blank control (Seo et al., 2015).


Inhibitionrate(%)= (3)

$$\frac{\text{Colonyareaof}\,\mathrm{negative}-\mathrm{Colony}\,\text{areaoftreatment}}{\text{Colonyareaofnegative}}\times 100$$

### 2.9 Statistical analysis of experimental data

Experimental data were analyzed using Excel (Microsoft, Redmond, WA, USA) and SPSS 24.0 software (IBM, Armonk, NY, USA) with experiments repeated three times. Data are reported as mean ± standard deviation (SD). Normality was tested by Shapiro–Wilk test and homogeneity of variances by Levene’s test. Colony diameters were measured by the cross method, and colony areas were quantified using ImageJ. Independent-samples *t*-test, one-way or two-way ANOVA followed by Duncan’s multiple range test were used for comparisons. A significance level was set at *p* ≤ 0.05 (Arifin, 2017).

## 3 Results

### 3.1 The identification of strain TRM68247

Strain TRM68247 was identified as *S. rochei* through morphological and molecular biological analyses. When streak-inoculated on Gauze’s Synthetic Medium No. 1, the strain displayed typical white or slightly yellowish *Streptomyces* colonies (Figure 1A), with well-developed aerial mycelium showing a villous or flocculent appearance. The substrate mycelium penetrated the medium, producing pigments, with characteristic visibility on the colony’s reverse side. The individual colonies displayed a flat and nearly circular morphology. Upon electron microscope examination, the spore chains were found to be organized in regular spirals, with spores appearing oval or cylindrical, featuring a smooth surface or fine wrinkles, as illustrated in Figure 1B. The construction of a phylogenetic tree using 16S rDNA sequences revealed a close evolutionary relationship between this strain and *S. rochei* NRRL B - 2410^T^ (Figure 1C), showing a high sequence similarity of 99.56%. Thus, strain TRM68247 was conclusively identified as *S. rochei.*

**FIGURE 1:**
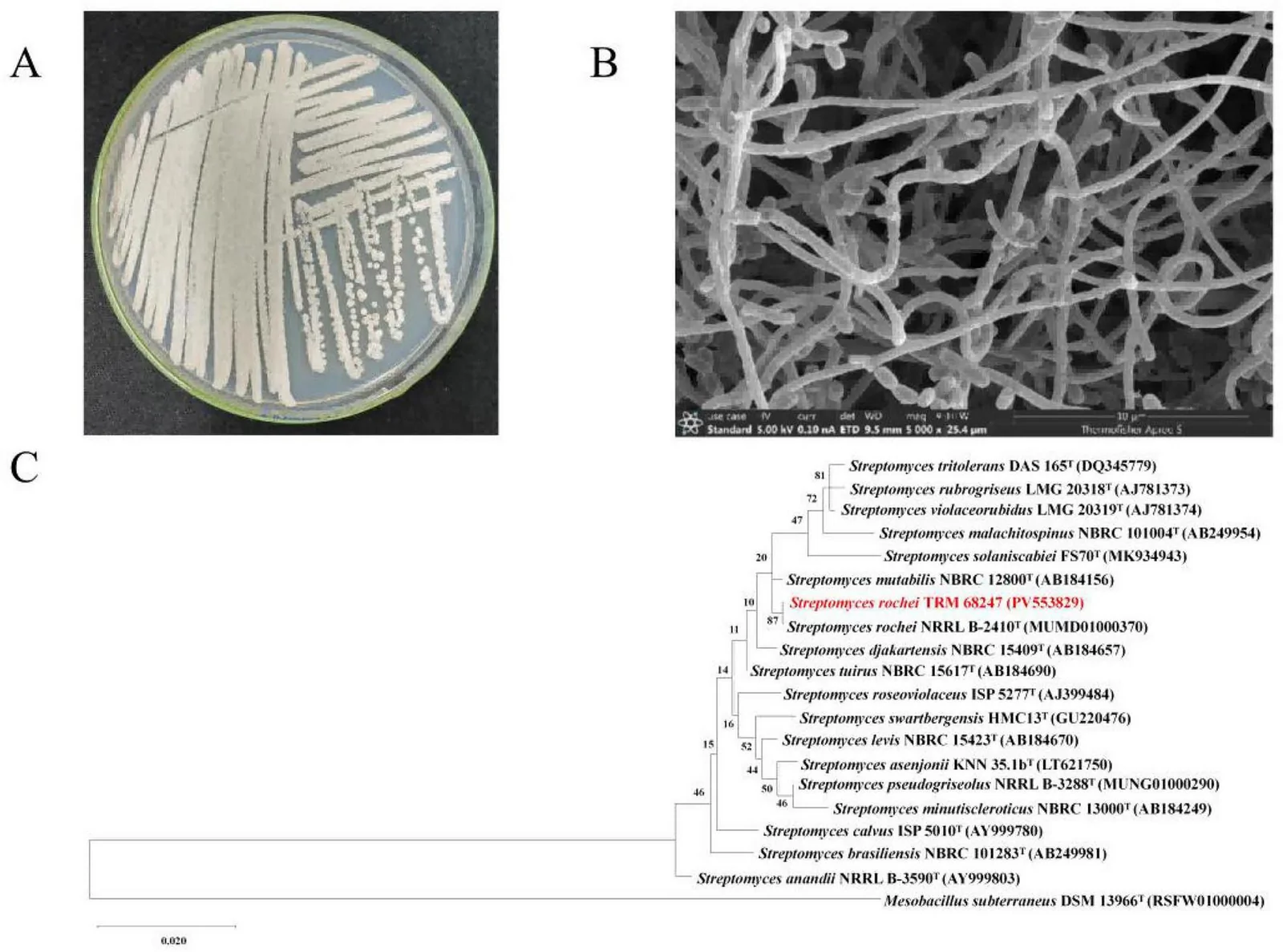
Morphological identification and phylogenetic analysis of strain TRM68247. **(A)** Strain TRM68247 on streak - inoculated Gauze’s Synthetic Medium No. 1. **(B)** SEM image of TRM68247 at 5,000 × magnification, showing regular spiral spore chains and oval/cylindrical spores with smooth surfaces or fine wrinkles. **(C)** Phylogenetic tree constructed from 16S rDNA sequences, illustrating the evolutionary relationships between TRM68247 and other Streptomyces species.

### 3.2 Genomic analysis of strain TRM68247: insights into genetic characteristics

#### 3.2.1 Genomic core characteristics and phylogenetic relationship analysis

Comprehensive analysis of the genomic characteristics and phylogenetic relationships of strain TRM68247 revealed significant genomic advantages and a clear evolutionary status, providing an important genetic basis for functional exploration and application potential evaluation of this strain.

Specifically, TRM68247 contained 7,074 genes, with a genome size of 8,114,439 bp, showing rich genetic diversity compared with 18 closely related strains. Compared with *S. rochei* NRRL-B 2410^T^, TRM68247 exhibited superior genome continuity with one-tenth of the contig count, 0.10% higher GC content indicating enhanced genomic robustness, 318 more genes, and a 432,038 bp longer genome, and the functions of the clusters are mainly focused on metabolism (Figure 2), suggesting expanded metabolic pathways and environmental adaptive capabilities.

**FIGURE 2:**
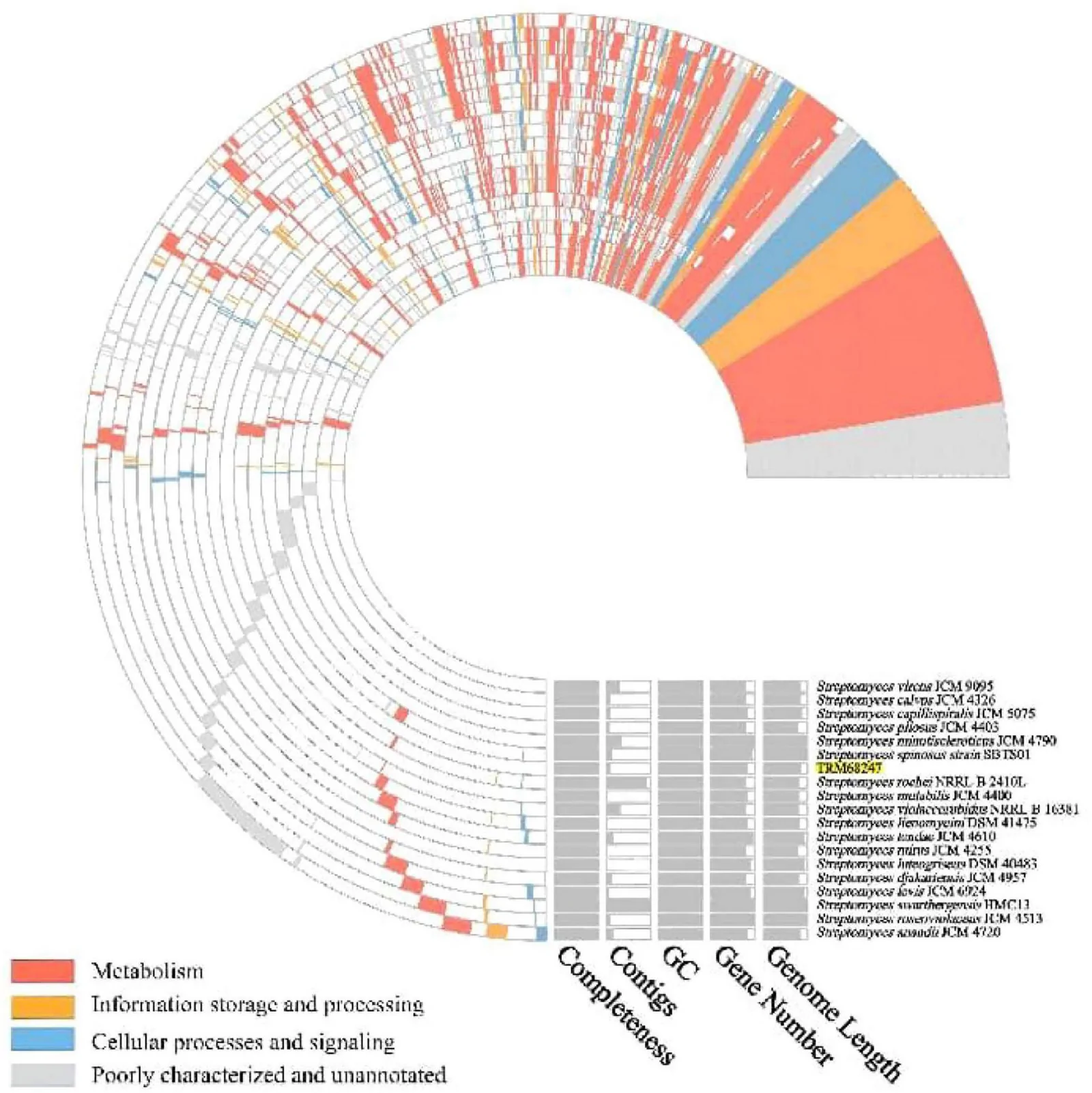
Visualization of genomic indicators of strain TRM68247 and its similar strains in a circular chart.

Furthermore, phylogenetic analysis based on ANI values showed that TRM68247 had a close evolutionary relationship with *S. rochei* NRRL B-2410L^T^, with an ANI value of 99.17% exceeding the 96% species delineation threshold, confirming their conspecificity. In contrast, the ANI value between TRM68247 and *Streptomyces mutabilis* JCM 4400^T^ was 90.64%, below the 96% threshold, indicating distinct species. The coherence of strains with ANI ≥ 96% and their clustering in the whole-genome phylogenetic tree further reinforced the reliability of the phylogenetic analysis (Figure 3).

**FIGURE 3:**
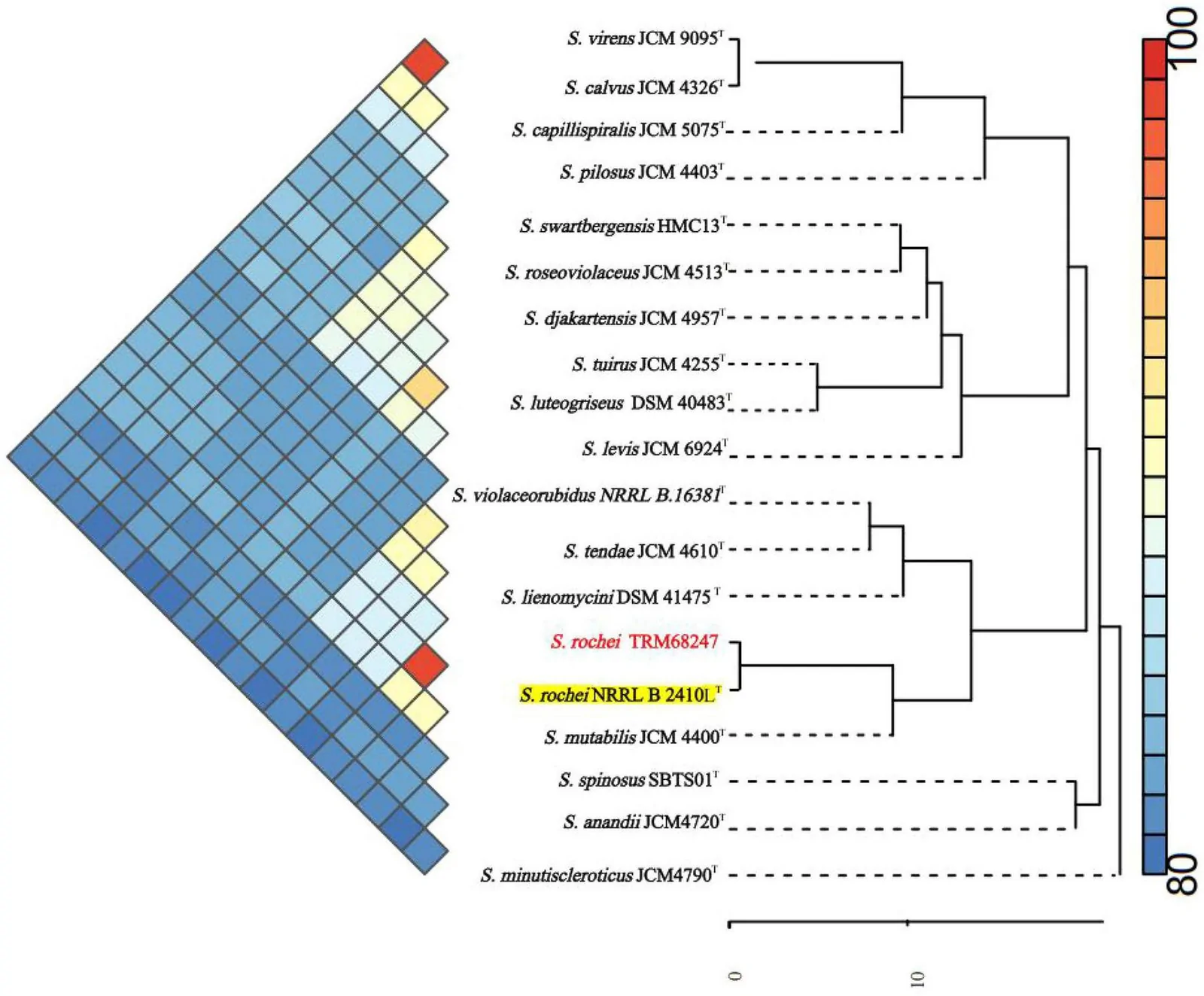
Comparative analysis of strain TRM68247 and similar strains using whole-genome sequences.

In conclusion, the excellent genomic characteristics and clear evolutionary status of TRM68247, as well as its close evolutionary relationship with *S. rochei* NRRL B - 2410L^T^, which confirms their conspecificity, lay a solid foundation for subsequent studies on its biocontrol function and metabolic potential.

#### 3.2.2 Pan-genome characteristics and mobile genetic elements function analysis

Pan-genome can analyze the evolutionary trend of similar strains as open, while horizontal gene transfer (HGT) facilitates the expansion of the prokaryotic gene pool by enabling microorganisms to acquire new functions through mobile genetic elements (MGEs) for better adaptation to diverse and extreme environments. Systematic analysis of the pan-genome characteristics and MGEs function of TRM68247 proved that combined contribution for the strain’s environmental adaptability and biocontrol function.

Pan-genome analysis was performed using the IPGA platform, and the results showed that the pan-genome of TRM68247 and its related strains contained 35,254 protein-coding genes, including 2,424 core genes, the core genome fitting curve equation derived from the power-law regression function is y = 5746.07 ± 52.95x^−0.33^ 0.01, R2 = 0.79. The pan-genome fitting curve equation is y = 6726.45 ± 64.17x^0.56^ 0.01, R2 = 0.97. The results are illustrated in Figure 4. The core genome was in a closed state. The fitting curve tended to be stable, ensuring the basic life activities of the strain. Meanwhile, the pan-genome was in an open state. The fitting curve increased with the increase of the genome number, endowing the strain with environmental adaptability and forming a “conservative-open” dual genomic structure.

**FIGURE 4:**
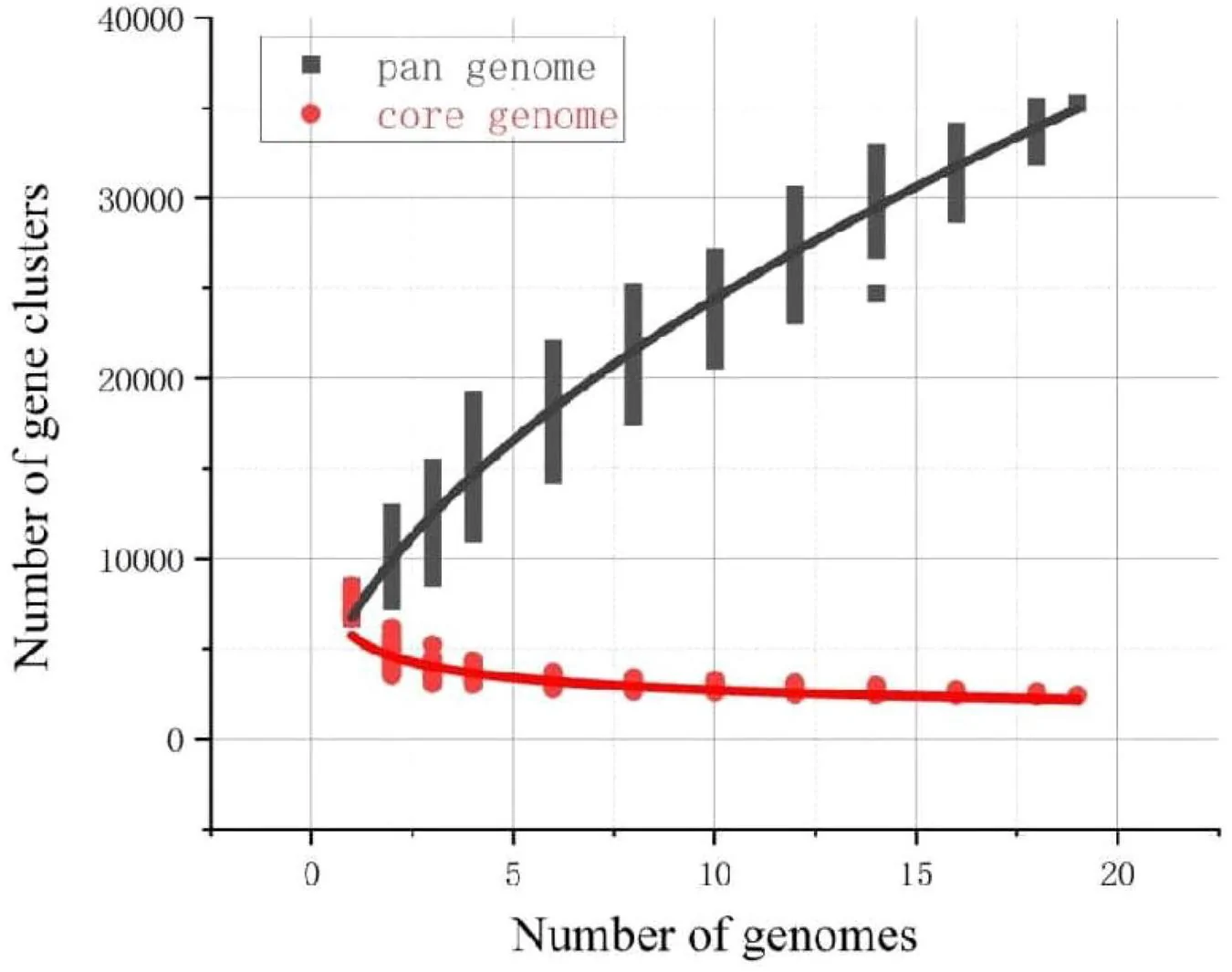
The pan-genome fitting curve of strain TRM68247.

A total of 15 insertion sequence (IS) elements belonging to five families were identified in the TRM68247 genome, with the IS 5 family being the most prevalent (12 sites), followed by IS 110, IS 256, and IS 701 families. Four plasmids with sizes ranging from 6.3 to 110 kb were also identified, among which plasmid No. 3011 (110 kb) may carry complex functional genes. In addition, three prophage regions were found. These MGEs significantly enhance genomic plasticity through HGT, helping the strain adapt to the arid environment of the Taklamakan Desert in Xinjiang (China) and optimize metabolic capacity (Supplementary Table 1).

In conclusion, the open pan-genome and MGEs jointly indicate enables TRM68247 to not only maintain core genetic characteristics but also achieve evolutionary innovation by acquiring exogenous functional genes, laying an important genetic foundation for maintaining the stability of its biocontrol function and the diversity of its metabolites.

### 3.3 Metabolic potential assessment of TRM68247

#### 3.3.1 Prediction of secondary metabolite BGCs

A statistical analysis was conducted on the natural product gene clusters of strain TRM68247 to investigate its metabolic capabilities. Supplementary Table 2 displays that TRM68247 harbors 47 natural product BGCs, categorized into 19 types. These encompass 1 lassopeptide, 3 RiPP-like, 3 NI-siderophore, 5 NRPS, 6 T1PKS, and 1 each of hydrogen-cyanide, lanthipeptide-class-i, melanin, furan, lanthipeptide-class-v, T3PKS, NRP-metallophore, NRPS, and ectoine. Lanthipeptide-class-iii is represented by 2 clusters, terpene by 6, NRPS-like by 3, T2PKS by 2, indole by 2, and hglE-KS, T1PKS, NRPS-like, and NRPS by 1 each. The diversity of these cluster types suggests the extensive metabolic potential of the strain and its diverse genetic basis for natural product synthesis, although the actual production and function of the corresponding metabolites remain to be verified experimentally.

Some gene clusters in TRM68247 display low similarity to established metabolite-synthesizing gene clusters, such as the NI-siderophore gene cluster (13% similarity to paulomycin), the NRPS gene cluster (6% similarity to A54145), and the furan gene cluster (9% similarity to methylenomycin A). Conversely, specific gene clusters exhibit high similarity to known products, such as the lanthipeptide-class-iii gene cluster (100% similarity to *SapB*), the terpene gene cluster (100% similarity to hopene), and the T3PKS gene cluster (100% similarity to flaviolin/1,3,6,8-tetrahydroxynaphthalene), suggesting that TRM68247 has the genetic potential to synthesize known or similar compounds; the actual production of these metabolites and their linkage to the corresponding clusters remain to be verified experimentally.

Strain TRM68247 exhibits considerable potential for enhancing plant growth and preventing diseases, particularly in Korla fragrant pear trees, based on its genome-encoded biosynthetic repertoire. The strain harbors multiple NI-siderophore gene clusters. Notably, an NI-siderophore gene cluster shares 100% similarity with the desferrioxamin B/desferrioxamine E gene cluster, suggesting that the strain may produce siderophores capable of chelating iron ions in the soil. Such siderophore production could improve the iron uptake and competitiveness of the strain in the soil and might potentially improve iron assimilation in Korla fragrant pear trees, thereby possibly enhancing their physiological functions and stress resistance; these plant-level effects require experimental validation. The ectoine gene cluster, exhibiting 100% similarity to the ectoine biosynthetic cluster, may facilitate the growth and survival of the strain in soil, and ectoine-related byproducts may have the potential to benefit Korla fragrant pear trees. Several NRPS (non-ribosomal peptide synthetase) and NRPS-like gene clusters were detected. These clusters are related to the biosynthesis of non-ribosomal peptide antibiotics and may endow the strain with antifungal activity for disease suppression. A gene cluster with 45% similarity to the lipopeptide 8D1-1/lipopeptide 8D1-2 gene cluster suggests a potential function in inhibiting pathogens of Korla fragrant pear. Moreover, T1PKS, T2PKS, and T3PKS gene clusters identified in the strain are predicted to synthesize antifungal polyketides, which may help control pear tree diseases. These predictions are based solely on genome mining and require direct experimental evidence to confirm the production and biological functions of the corresponding metabolites.

Notably, *S. rochei* TRM68247 possesses a genetic basis for synthesizing diverse VOCs. Among all predicted secondary metabolite biosynthetic gene clusters, 7 VOC-related gene clusters were identified, which are mainly involved in three major metabolic pathways: terpenes, indoles, and furans. Among them, terpene-type gene clusters contribute most significantly to VOC biosynthesis. Specifically, the strain contains four functionally annotated terpene gene clusters, including one with 100% homology to the hopene biosynthetic cluster, one with 100% homology to the albaflavenone biosynthetic cluster, and two terpene clusters associated with isorenieratene biosynthesis. These clusters are predicted to encode volatile terpenes such as hopene and albaflavenone. Meanwhile, the strain also harbors 2 indole-type gene clusters and 1 furan-type gene cluster. The two indole clusters show 100% and 83% homology, respectively, and are predicted to be involved in the biosynthesis of odorous indole derivatives. The furan-type cluster shares 9% homology with methylenomycin A, and its metabolites may participate in the formation of volatile substances. In addition, there are some terpene gene clusters with incompletely annotated functions in the genome. Although their specific products remain to be verified, they also provide important genetic potential for VOC biosynthesis. Direct experimental evidence linking these clusters to the VOCs detected by SPME-GC-MS (e.g., gene deletion or heterologous expression) is still required.

In summary, the genome of *S. rochei* TRM68247 encodes at least 4 terpene-type VOC biosynthetic gene clusters with high sequence homology to characterized clusters, together with multiple indole- and furan-related clusters, indicating the strong genetic potential of this strain to synthesize complex volatile metabolites. Given the predicted antifungal activity of several VOC-related BGCs, TRM68247 warrants further investigation as a promising candidate for promoting growth and disease resistance in Korla fragrant pear trees and other plant species, subject to experimental validation.

#### 3.3.2 Identification of potential drug resistance gene in *S. rochei* TRM68247

Analysis of the genome of *S. rochei* TRM68247 against the CARD revealed the presence of two antibiotic resistance genes: *mgtA* (macrolide glycosyltransferase) and *otr(A)* (tetracycline resistance ribosome protection protein). These genes confer resistance to macrolide and tetracycline antibiotics, respectively, suggesting that the strain may possess the capacity to tolerate these antibiotics, potentially as part of a self-protection mechanism linked to antibiotic production, consistent with the co-evolutionary relationship between resistance genes and antibiotic production in *Streptomyces* (Table 1); this inference requires experimental verification. *mgtA* may contribute to macrolide resistance through glycosylation-mediated modification, whereas *otr(A)* is associated with tetracycline resistance via ribosome protection.

**TABLE 1:** High-throughput resistance profiling of *Streptomyces rochei* TRM68247 against comprehensive antibiotic resistance database.

RGI criteria	ARO term	Detection criteria	AMR gene family	Drug class	Resistance mechanism	Identity of matching region	Length of reference sequence
Strict	mgtA	Protein homolog model	Mgt macrolide glycotransferase	Macrolide antibiotic	Antibiotic inactivation	86.89%	98.56%
Strict	*Streptomyces lividans* otr(A)	Protein homolog model	Tetracycline -resistant ribosomal protection protein	Tetracycline antibiotic	Antibiotic target protection	85.21%	98.75%

### 3.4 Evaluation of VOCs from strain TRM68247 for biocontrol activity

#### 3.4.1 Temporal and spatial inhibitory effects of VOCs from TRM68247

Studies on the antifungal properties of VOCs released by *S. rochei* TRM68247 showed that, compared with the control group, the growth of *V. pyri* and *A. gaisen* in Petri dishes fumigated with VOCs was significantly inhibited, and the inhibitory effect exhibited obvious temporal and spatial differences with the extension of culture time. It can be seen from the colony morphology in Figure 5 that the expansion rate of pathogenic colonies in the treatment group was significantly slower than that in the control group, and the colony density was also significantly reduced, while the colonies in the control group grew vigorously with intact morphology, which intuitively confirmed the antifungal activity of VOCs from *S. rochei* TRM68247.

**FIGURE 5:**
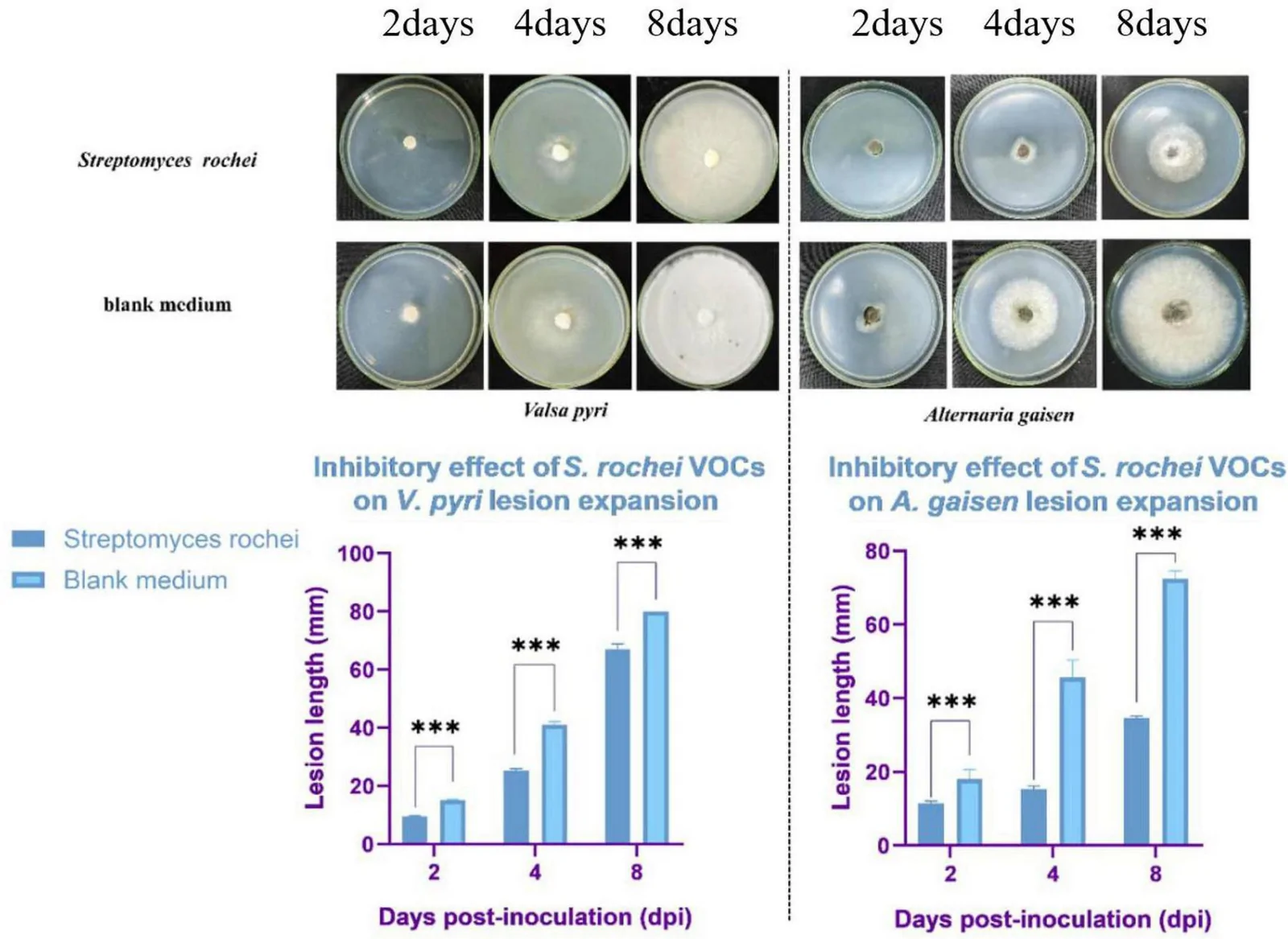
Spatiotemporal inhibitory effect of *S. rochei* VOCs on mycelial growth of *V. pyri* and *A. gaisen*. Colony morphology and lesion length statistics of *V. pyri* (left) and *A. gaisen* (right) under VOC treatment (*S. rochei*) and control (blank medium) at 2, 4, and 8 days post-inoculation (dpi). Data are presented as mean ± SD (*n* = 3). Normality was tested by Shapiro–Wilk test and homogeneity of variances was tested by Levene’s test before statistical analysis. Differences between the treatment and control groups at each time point were analyzed using independent samples *t*-test. *** Indicates extremely significant difference between the treatment and control groups at the same time point (*p* < 0.001).

Combined with the analysis of lesion length statistics in Figure 5, there were differences in the sensitivity of the two pathogens to VOCs from *S. rochei* TRM68247 and the temporal dynamics of the antifungal effect. Among them, the antifungal effect on *V. pyri* showed a trend of first increasing and then decreasing, with strong antifungal activity in the early stage; the antifungal effect was better on 2 and 4 d, but significantly decreased on 8 d. *A. gaisen* was more sensitive to VOCs from strain TRM68247, and the overall antifungal effect was better than that on *V. pyri*. Its antifungal effect showed a trend of first significantly increasing and then slightly decreasing with the culture time: a certain antifungal effect was observed on 2 days, the antifungal effect reached the peak on 4 days, and although it slightly decreased on 8 days, it still maintained a relatively high level, indicating that VOCs from strain TRM68247 had a continuous and stable inhibitory effect on *A. gaisen*.

In conclusion, the VOCs produced by *S. rochei* TRM68247 exerted remarkable inhibitory effects on the mycelial growth of *V. pyri* and *A. gaisen*. However, the long-term persistence of this antifungal activity was limited; in particular, the inhibitory efficacy against *V. pyri* declined markedly at the later stage.

#### 3.4.2 Biocontrol activity of VOCs from *S. rochei* TRM68247 based on *in vitro* materials

##### 3.4.2.1 Evaluation of control measures for pear tree canker on young shoots

Young pear shoots were utilized to assess the efficacy of VOCs produced by *S. rochei* strain TRM68247 against pear tree canker. The results showed that VOCs released by TRM68247 could significantly inhibit the lesion expansion of *V. pyri*. The control efficacy reached 49.33% and 74.91% at 7 and 14 days after treatment, respectively, which was significantly higher than that of the blank control group. It was confirmed that this strain possessed favorable antagonistic activity and biocontrol application potential.

With the extension of treatment time, lesions in the blank control group expanded rapidly, accompanied by severe epidermal browning and rot, as well as obvious deterioration of phloem and subcutaneous tissues (Figure 6). As shown in Table 2, lesion development was markedly restricted in the TRM68247 treatment group, and the tissue integrity of pear shoots was well maintained. Its control effect was comparable to the positive control eugenol group, which exhibited control efficacies of 66.36% and 91.10% at 7 and 14 days, respectively. Although the lesion severity increased over time in all groups, the lesion expansion rate in the TRM68247 group was consistently significantly lower than that in the blank control. Meanwhile, its control efficacy gradually increased with time, which could effectively delay the occurrence and development of the disease and displayed a good long-term biocontrol performance.

**FIGURE 6:**
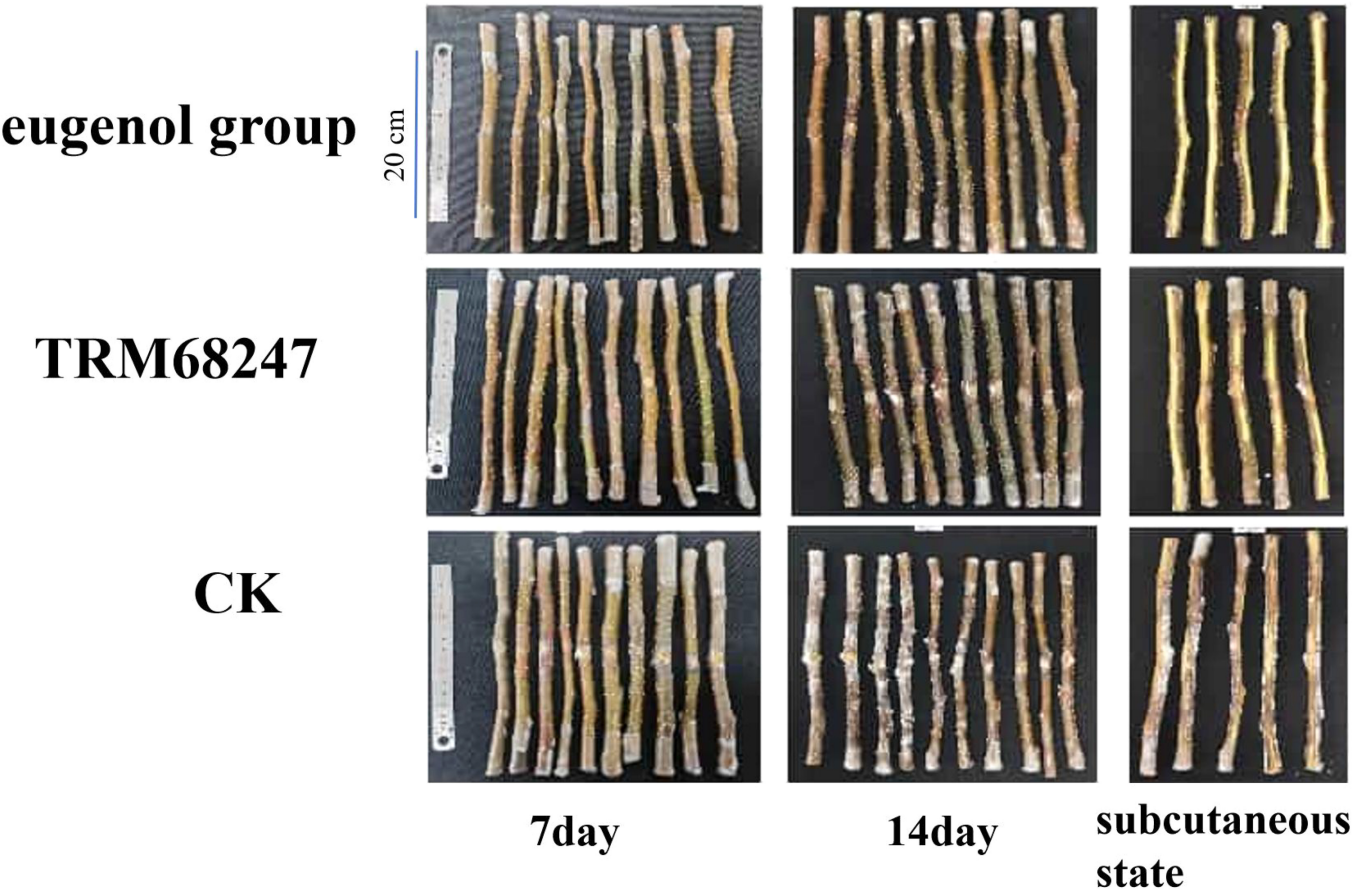
Efficacy of fumigation treatments in controlling pear tree canker on young pear shoots. Representative images of lesion development on pear shoots at 7 and 14 days post-treatment, as well as the subcutaneous tissue status after longitudinal sectioning. CK, blank control group; Eugenol, positive control group.

**TABLE 2:** Effects of different treatments on pear tree canker control.

Treatment	7 days		14 days	
	Lesion length (cm)	Control efficacy %	Lesion length (cm)	Control efficacy %
Eugenol	8.04 ± 1.51^c^	66.36	9.93 ± 2.72^c^	91.10
TRM68247	12.11 ± 2.84^b^	49.33	27.98 ± 7.12^b^	74.91
CK	23.90 ± 5.81^a^	–	11.53 ± 23.44^a^	–

Values are presented as mean ± SD (*n* = 3). Normality was tested by Shapiro–Wilk test and homogeneity of variances was tested by Levene’s test before analysis. Different lowercase letters in the same column indicate significant differences at *P* < 0.05. After verifying normality (Shapiro–Wilk test) and homogeneity of variance (Levene’s test), one-way ANOVA followed by Duncan’s multiple range test was performed to compare differences among treatments at each time point. CK represents the blank control group; Eugenol serves as the positive control group.

As shown in Table 2, the VOCs of *S. rochei* TRM68247 could maintain a stable inhibitory effect throughout the experimental period. Although its overall biocontrol efficiency was slightly lower than that of eugenol, it still presented strong potential for practical application.

##### 3.4.2.2 Efficacy of control measures for managing pear black spot on fruit

The control efficacy of various fumigation treatments against Alternaria black spot, caused by *A. gaisen* on mature fragrant pear fruits was systematically evaluated in this study. The results revealed that VOCs originating from *S. rochei* TRM68247 successfully inhibited the development of Alternaria black spot on pear fruits, surpassing the control efficacy of eugenol.

Disease phenotypes of fragrant pear fruits varied significantly across treatment groups, as depicted in Figure 7. The blank control group displayed severe symptoms, including large dark lesions, evident epidermal damage, and deep internal infection in wounded and inoculated fruits. Extensive browning and rot were evident in the pulp tissues, with indistinct boundaries between healthy and rotten tissues. Eugenol treatment, serving as the positive control, significantly mitigated disease severity relative to the blank control; however, minor lesions on the fruit surface and pulp browning persisted. Disease progression in the eugenol group was significantly less severe than in the blank control group but remained numerically higher than in the TRM68247 treatment group; the difference between the two treatments was not statistically significant (Table 3). Fruits exposed to VOCs from strain TRM68247 exhibited the lowest disease severity, with minimal superficial lesions and an intact epidermis, although they did not differ significantly from the eugenol-treated fruits in surface lesion size or internal infection depth (Table 3).

**FIGURE 7:**
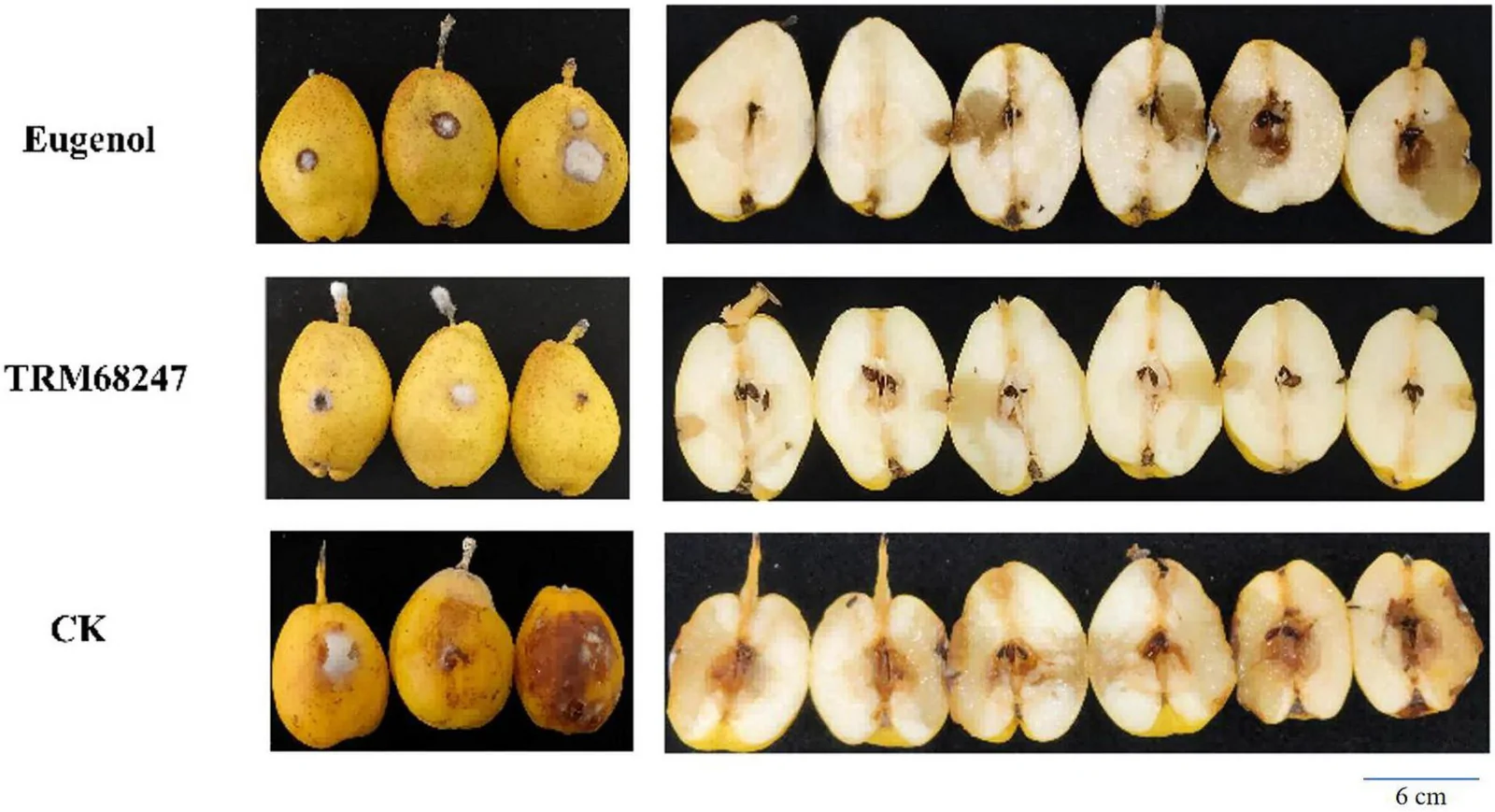
Control efficacy of different fumigation treatments against *Alternaria* black spot on mature fragrant pear fruits. Fruits were inoculated with *A. gaisen* and treated with CK (blank control), Eugenol (positive control), and TRM68247 (VOCs of *S. rochei* TRM68247). For each treatment, whole fruits **(left)** and corresponding longitudinal sections **(right)** are shown to compare lesion development.

**TABLE 3:** Effects of various treatments on *Alternaria* black spot infection of mature fragrant pear fruits.

Treatment	Lesion size on pear surface /L (mm)		Infection depth of pear internal section/d (mm)	
	Lesion length	Control efficacy %	Infection depth	Control efficacy %
Eugenol	15.40 ± 5.80^b^	72.97	20.80 ± 8.55^b^	57.64
TRM68247	11.97 ± 1.17^b^	78.99	15.17 ± 5.39^b^	69.11
CK	56.96 ± 26.75^a^	–	49.11 ± 14.22^a^	–

Data are expressed as mean ± SD (*n* = 3). Normality was tested by Shapiro–Wilk test and homogeneity of variances was tested by Levene’s test before analysis. Different lowercase letters in the same column indicate significant differences according to Duncan’s multiple range test at *P* < 0.05. After verifying normality (Shapiro–Wilk test) and homogeneity of variance (Levene’s test), one-way ANOVA followed by Duncan’s multiple range test was performed to compare differences among treatments. CK represents the blank control group; control efficacy was not calculated for CK.

According to the quantitative results in Table 3, there were significant differences in surface lesion size and internal infection depth of fragrant pear fruits among all treatments. The control efficacy of eugenol against surface black spot was 72.97%, and its control effect on internal infection was 57.64%. By comparison, the VOCs from strain TRM68247 achieved a surface control efficacy of 78.99% and an internal infection control efficacy of 69.11%, which were significantly higher than those of the eugenol treatment. The TRM68247 treatment could effectively reduce the surface lesion size and inhibit the inward spread of pathogens. All lesion-related indicators were markedly better than those of the eugenol and blank control groups, showing the optimal control efficacy both on the fruit surface and in internal tissues.

Combined with the phenotypic observation in Figure 7 and the quantitative data in Table 3, it was further confirmed that the antifungal and disease control effects differed significantly among treatments. This indicates that VOCs derived from strain TRM68247 possess superior biocontrol potential and disease control efficacy against *Alternaria* black spot of fragrant pear.

### 3.5 Detection and analysis of antagonistic VOCs from TRM68247

#### 3.5.1 Analysis of the VOCs components in TRM68247

To clarify the component characteristics and potential antifungal activity of VOCs emitted by *S. rochei* TRM68247, we performed SPME-GC-MS analysis. With blank Gauze’s Synthetic Medium No. 1 as the control, a total of 44 VOCs were identified. Among them, eight components with relative contents higher than 1% were defined as main VOCs, and 5 of them were screened as potential active substances.

To clarify the component characteristics and potential antibacterial activity of VOCs emitted by *S. rochei* TRM68247, the VOCs were analyzed through SPME-GC-MS in this study, resulting in the identification of 44 VOCs. Among them, eight VOCs with a content exceeding 1% were classified as main VOCs, and five of these were identified as potential active VOCs.

Specifically, a total of 44 VOCs were identified with a mass spectral library match degree ≥ 80%, spanning various categories, including 14 hydrocarbons, 10 alcohols, 10 silicon-containing compounds and other substances, as well as 4 esters, 3 ketones, 1 ether, 2 acids, and 1 phenolic compound. The identified VOCs are detailed in Supplementary Table 2. Notably, three compounds, namely hexamethylcyclotrisiloxane(24.20%), cis- 5 -methyl-2- (1-methylethyl) cyclohexanone (19.86%), and 3,5-di-tert-butylphenol (16.55%), had contents exceeding 10% and were classified as core high-abundance components, collectively constituting over 60% of the total VOCs. Additionally, eight VOCs exhibited contents greater than 1%, such as 2-ethyl-1-hexanol and nonanal, and these eight compounds were regarded as the main VOCs components. The specific structures of these components are illustrated in Figure 8.

**FIGURE 8:**
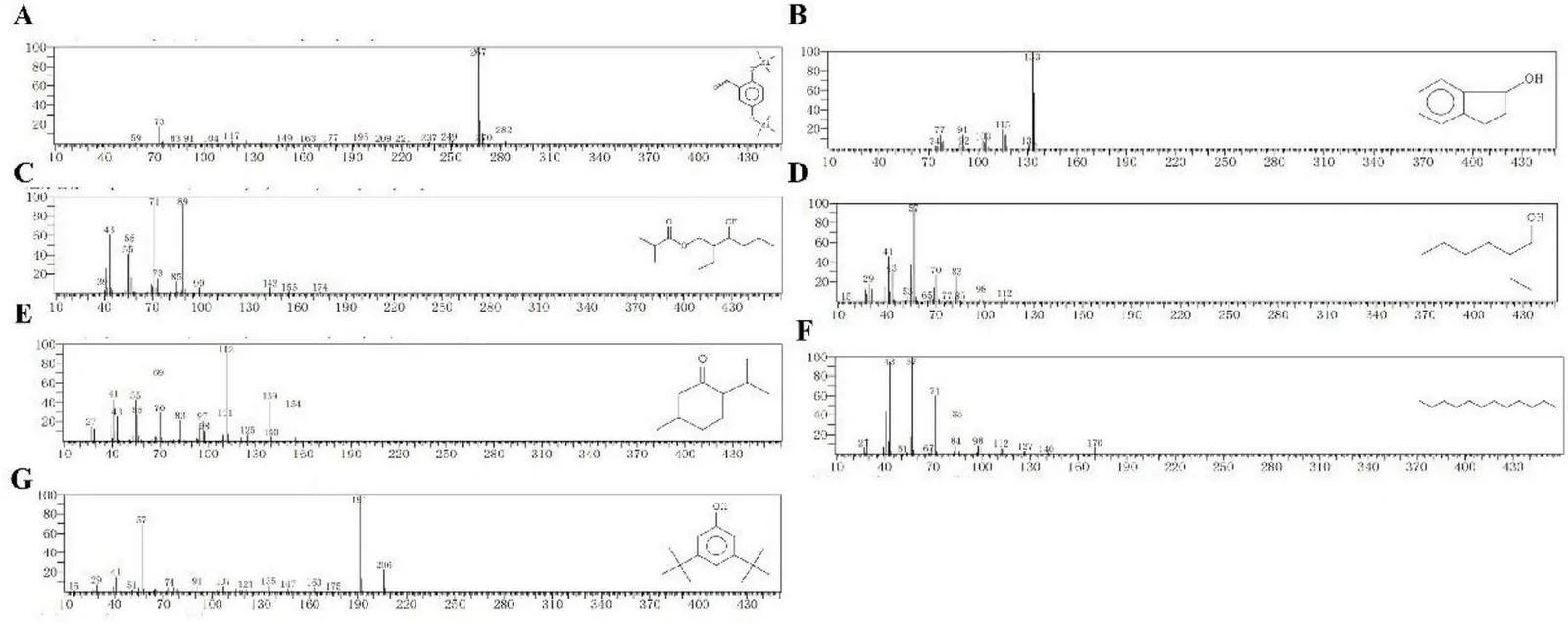
Mass Spectrometry Identification Spectra of Main VOCs from Strain TRM68247. **(A)** 2-methylpropanoate. **(B)** 2,3-dihydro- 1H-inden -1-ol. **(C)** Nonanal. **(D)** 2-ethyl-1-hexanol. **(E)** Isomenthone. **(F)** Dodecane. **(G)** 3,5-Di-tert-butylphenol. Mass spectral characteristic peaks corresponding to the top seven VOCs with the highest relative contents in strain TRM68247. The chemical structures of each compound are shown on the right.

Through a comprehensive literature review and analysis of compound structure, 5 out of the 8 major volatile components produced by strain TRM68247 have been identified as potential antifungal agents, namely 3,5-di-tert-butylphenol, nonanal, 2-ethyl-1-hexanol, isomenthone, and 2,3-dihydro-1H-inden-1-ol. The biocontrol effect of strain TRM68247 against *V. pyri* and *A. gaisen* may be attributed to the individual effects of these active substances.

#### 3.5.2 Active substances of VOCs

The study utilized the divided plate method in conjunction with a 10-fold serial dilution assay to systematically assess the volatile antifungal activity of five compounds, namely nonanal, 2,3-dihydro-1H-inden-1-ol, 2-ethyl-1-hexanol, 3,5-di-tert-butylphenol, and isomenthone, against *V. pyri*. and *A.gaisen*. The results showed that the five tested compounds exhibited distinct differences in their antifungal responses to the two pathogens. Among them, 2-ethyl-1-hexanol and 3,5-di-tert-butylphenol displayed relatively prominent antifungal potential, both exerting inhibitory effects on the two fungal pathogens to varying degrees.

As shown in Figure 9 and Table 4, 2-ethyl-1-hexanol exhibited the strongest volatile antifungal activity against *V. pyri*, with a VMIC of 5 × 10^−1^ μg⋅mL^(1^. At this concentration, the inhibition rate reached 96.41%, and it increased to 97.47% at 5 × 10^2^ μg⋅mL^(1^, achieving nearly complete inhibition of mycelial growth. The VMIC of 3,5-di-tert-butylphenol was 5 × 10^2^ μg⋅mL^–1^, with an inhibition rate of 96.05% at this concentration. Although it exerted prominent antifungal effects at high concentration, its activity declined rapidly as the concentration decreased, and the inhibition rate dropped to 70.12% at 5 × 10^1^ μg⋅mL^–1^; even a growth-promoting effect was observed at low concentrations. In comparison, 2,3-dihydro-1H-inden-1-ol and isomenthone only showed moderate antifungal effects at medium and high concentrations, with the maximum inhibition rates of 52.07% and 47.04%, respectively. Nonanal had no inhibitory effect on *V. pyri*, and negative inhibition rates were recorded at most concentrations, indicating an obvious growth-promoting effect (Figure 9 and Table 4).

**FIGURE 9:**
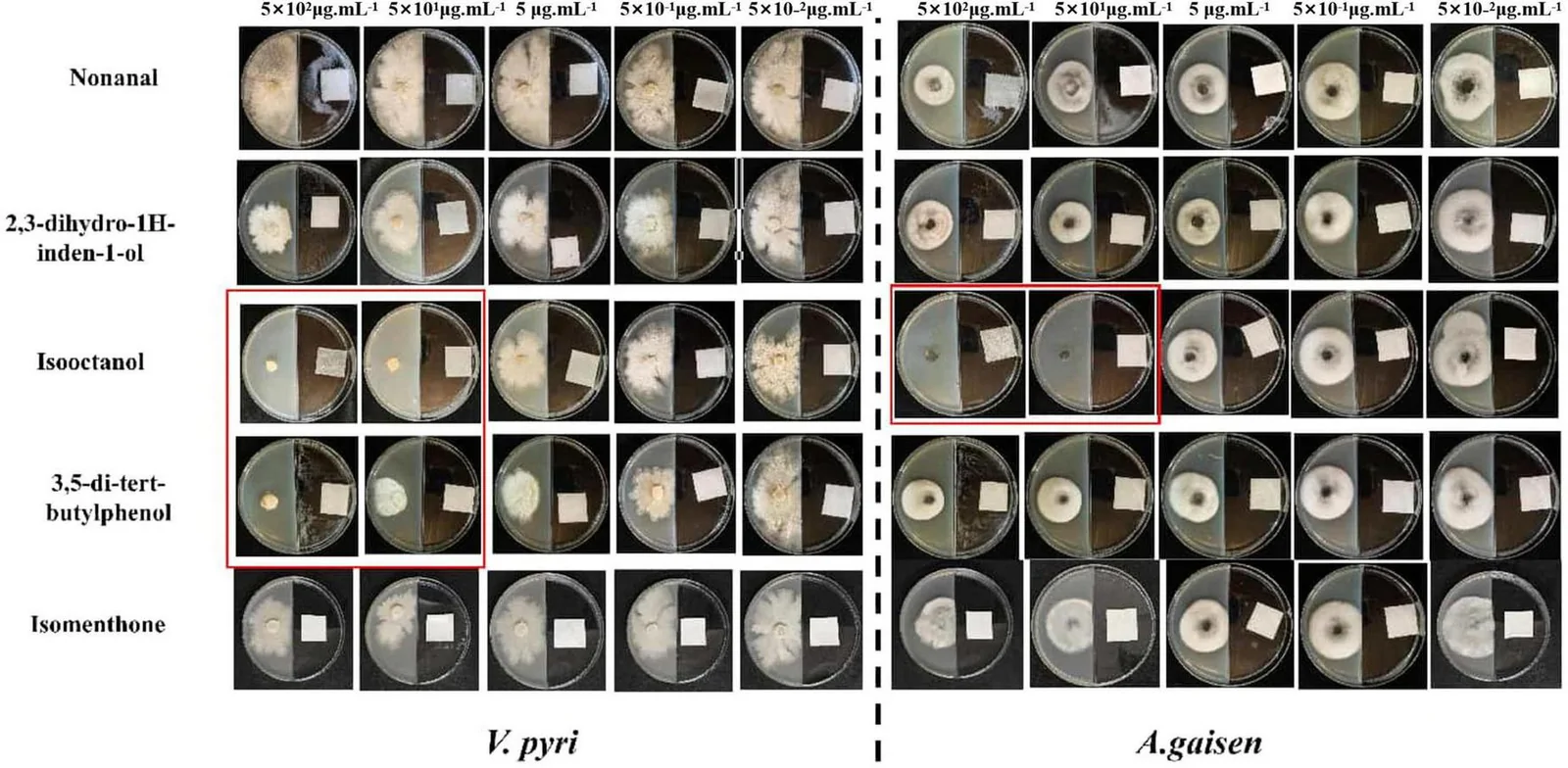
Volatile antifungal activity of five VOCs produced by strain TRM68247 against *V. pyri* (left panel) and *A. gaisen* (right panel) determined via dual-compartment plate confrontation assay. All pathogens were cultured on PDA medium at 28 °C for 7 days, with five 10-fold serial concentrations (5 × 10^2^ to 5 × 10-2 μg⋅mL^(1^) set for each compound, and pure methanol was used as the blank control.

**TABLE 4:** Volatile antifungal activity of five compounds against *V. pyri* and *A. gaisen*.

Pathogen	Compound	Index	Various treatment concentrations (μ g⋅mL^–1^)				
			5 × 10^2^	5 × 10^1^	5	5 × 10^(1^	5 × 10^–2^
*V. pyri*	Nonanal	Colony area (mm^2^)	1924.26 ± 4.00^aA^	1924.18 ± 1.15^aA^	1900.36 ± 33.54^aA^	1521.55 ± 28.34^aC^	1756.14 ± 13.17^aB^
		Inhibition rate (%)	-11.62	-11.61	-10.23	11.74	-1.86
	2,3-dihydro-1H-inden-1-ol	Colony area (mm^2^)	826.28 ± 2.27^cC^	829.42 ± 5.02^cC^	1136.64 ± 3.74^bB^	1142.03 ± 30.15^bB^	1646.92 ± 13.94^bA^
		Inhibition rate (%)	52.07	51.89	34.07	33.73	4.47
	2-ethyl-1-hexanol	Colony area (mm^2^)	43.65 ± 0.68^eD^	61.81 ± 0.28^eD^	1030.78 ± 4.13^cC^	1115.80 ± 2.86^bB^	1253.48 ± 19.51^dA^
		Inhibition rate (%)	97.47	96.41	40.21	35.28	27.28
	3,5-di-tert-butylphenol	Colony area (mm^2^)	68.02 ± 1.01^dE^	515.06 ± 5.31^dD^	755.05 ± 0.44^dC^	819.09 ± 0.73^cB^	1783.22 ± 2.62^aA^
		Inhibition rate (%)	96.05	70.12	56.2	52.49	−3.43
	Isomenthone	Colony area (mm^2^)	912.98 ± 6.84^bD^	994.04 ± 2.72^bC^	1182.53 ± 1.28^bB^	1177.45 ± 0.74^bB^	1441.81 ± 5.48^cA^
		Inhibition rate (%)	47.04	42.34	31.41	31.7	16.36
*A. gaisen*	Nonanal	Colony area (mm^2^)	552.05 ± 1.06^dD^	815.65 ± 5.26^bC^	853.84 ± 1.18^bC^	1126.81 ± 19.18^bB^	1318.97 ± 23.12^bA^
		Inhibition rate (%)	62.32	44.33	41.72	23.09	9.98
	2,3-dihydro-1H-inden-1-ol	Colony area (mm^2^)	810.27 ± 0.35^aBC^	604.79 ± 1.65^dD^	751.59 ± 17.32^cC^	862.63 ± 6.62^cB^	1327.59 ± 47.47^bA^
		Inhibition rate (%)	44.7	58.72	48.7	41.12	9.39
	2-ethyl-1-hexanol	Colony area (mm^2^)	59.69 ± 0.22^eC^	66.89 ± 1.48^eC^	965.46 ± 21.80^aB^	1370.32 ± 115.87^aA^	1528.84 ± 19.98^aA^
		Inhibition rate (%)	95.93	95.43	34.09	6.47	−4.35
	3,5-di-tert-butylphenol	Colony area (mm^2^)	612.83 ± 1.47^cD^	706.41 ± 1.29^cD^	919.35 ± 59.96^abC^	1061.14 ± 73.84^bB^	1285.23 ± 117.67^bA^
		Inhibition rate (%)	58.17	51.78	37.25	27.58	12.27
	Isomenthone	Colony area (mm^2^)	725.51 ± 3.05^bD^	962.97 ± 16.51^aBC^	938.57 ± 28.87^aC^	1097.29 ± 97.64^bB^	1261.16 ± 133.19^bA^
		Inhibition rate (%)	50.48	34.28	35.94	25.09	13.92

Data are expressed as mean ± SD (*n* = 3). Normality was tested by Shapiro–Wilk test and homogeneity of variances was tested by Levene’s test before analysis. The *V. pyri* and *A. gaisen* treated only with methanol without the addition of volatile compounds were set as the negative controls, with their colony areas determined as 1723.98 ± 7.35 mm^2^ and 1465.16 ± 52.06 mm^2^, respectively, which were used for the calculation of inhibition rate. Different uppercase letters in the same column indicate significant differences among different compounds at the same concentration, and different lowercase letters in the same row indicate significant differences among different concentrations of the same compound (*p* < 0.05). Two-way ANOVA followed by Duncan’s multiple range test was used for statistical analysis.

For *A. gaisen*, 2-ethyl-1-hexanol showed the strongest antifungal activity. Its VMIC was 5 × 10^−1^ μg⋅mL^−1^, at which colony growth was reduced by 1,398 mm^2^ (95.43%) relative to the untreated control (mean colony area 1465 mm^2^); at 5 × 10^2^ μg⋅mL^−1^ the reduction was 1,405 mm^2^ (95.93%), indicating nearly complete inhibition of pathogen growth (Figure 9 and Table 4). At the highest tested concentration (5 × 10^2^ μg⋅mL^−1^), 2-ethyl-1-hexanol reduced colony area by 492 mm^2^ more than nonanal (95% CI: 489.7–495.0 mm^2^) and by 553 mm^2^ more than 3,5-di-tert-butylphenol (95% CI: 549.5–556.8 mm^2^). Nonanal and 3,5-di-tert-butylphenol showed statistically significant inhibitory effects against *A. gaisen* only at 5 × 10^2^ μg⋅mL^−1^, reducing colony growth by 913 mm^2^ (62.32%) and 852 mm^2^ (58.17%), respectively. These values did not meet the VMIC criterion (≥95% inhibition), and the inhibitory effects declined rapidly as the concentration decreased. 2,3-dihydro-1H-inden-1-ol and isomenthone showed weak antifungal activity over the tested range, reducing growth by at most 860 mm^2^ (58.72%) and 739 mm^2^ (50.48%), respectively; neither compound achieved complete growth inhibition (Figure 9 and Table 4).

In summary, the five VOCs showed varying degrees of species selectivity in their antifungal efficacy against the pear pathogens *V. pyri* and *A. gaisen*. 2-ethyl-1-hexanol exhibited strong inhibitory effects against both pathogens, whereas 3,5-di-tert-butylphenol showed significant antifungal activity specifically against *V. pyri*. Nonanal and 3,5-di-tert-butylphenol exerted only limited inhibitory effects on *A. gaisen*, observable only at high concentrations; the remaining compounds showed weak or negligible activity against *A. gaisen*.

## 4 Discussion

Korla fragrant pear is a characteristic economic fruit in Xinjiang (China). During postharvest storage and transportation, the fruit is highly susceptible to infection by *V. pyri* and *A. gaisen*, which cause severe postharvest rot. These diseases deteriorate fruit quality, shorten shelf life and result in substantial economic losses to the industry. At present, chemical fungicides are still the dominant approach to control postharvest diseases of fragrant pear. However, long-term and excessive application of chemicals not only induces pathogen resistance, but also leads to pesticide residues and environmental pollution. Therefore, developing safe, long-acting and eco-friendly biological preservation and disease control technologies has become an urgent demand for the green development of the postharvest pear industry (Martínez-Espinosa, 2020).

Microorganisms inhabiting extreme environments have evolved unique metabolic pathways to adapt to harsh habitats and can synthesize a large number of novel natural products with excellent biological activities. They are regarded as important resources for exploring active substances for fruit postharvest preservation (Narayanan et al., 2025). In this study, *S. rochei* TRM68247 was isolated from the rhizosphere soil of *T. ramosissima* in the Taklimakan Desert. Its volatile organic compounds (VOCs) exhibited remarkable antagonistic effects against the two major postharvest pathogens of fragrant pear. Accumulated studies have fully demonstrated that *S. rochei*, as a classic biocontrol actinomycete, can inhibit a variety of phytopathogenic fungi and exert plant growth-promoting effects, showing great application potential in the prevention and control of fruit diseases (Nguyen et al., 2024).

The secondary metabolic synthesis capacity of microorganisms is determined by biosynthetic gene clusters (BGCs) in the genome, and genomic analysis can interpret the metabolic characteristics and environmental adaptability of strains at the genetic level (Cimermancic et al., 2014). The draft genome assembly of strain TRM68247 is 8.1 Mb in length, and a total of 47 secondary metabolic BGCs were annotated, which was significantly more than the 27 BGCs identified in the model strain *Streptomyces coelicolor* A3(2) (Schlimpert and Elliot, 2023). The average nucleotide identity (ANI) between TRM68247 and the type strain *S. rochei* NRRL B-2410T reached 99.17%, confirming that they belong to the same species. Meanwhile, TRM68247 possesses a larger genome and higher GC content, forming unique genetic characteristics. Fifteen insertion sequence (IS) elements belonging to five families were identified in its genome. Combined with its open pan-genome feature, horizontal gene transfer enhances the genomic plasticity of the strain, enabling it to survive in extreme desert environments and maintain the diversity of secondary metabolites. This characteristic also reflects the unique value of extremophilic actinomycetes in the development of biocontrol and preservation resources for postharvest fruits (Helmi, 2025).

AntiSMASH analysis revealed that TRM68247 contains 19 categories of secondary metabolic BGCs, covering nonribosomal peptide synthetases (NRPS), polyketide synthases (PKS), terpenoids and ribosomally synthesized and post-translationally modified peptides (RiPPs). Among them, seven BGCs were predicted to participate in VOC biosynthesis, mainly corresponding to terpenoid, indole and furan metabolic pathways, and four terpenoid BGCs shared high homology with sequences for synthesizing antifungal precursors. Annotation results from the Comprehensive Antibiotic Resistance Database (CARD) showed that the strain carried two antibiotic resistance genes, mgtA and otr(A), which conform to the co-evolution rule of antibiotic biosynthesis and self-resistance in streptomycetes. This finding genetically confirms that TRM68247 has the capacity to synthesize diverse antifungal active metabolites (Adamek et al., 2019). It should be noted that volatile components detected in this study, such as 2-ethyl-1-hexanol and 3,5-di-tert-butylphenol, are mostly derived from metabolic intermediates and cellular secretions, which do not have a one-to-one correspondence with typical secondary metabolites predicted by BGC analysis. To date, no BGCs specifically responsible for synthesizing these VOCs have been reported in relevant literature, and the correlation between them remains to be further explored (Dawid and Hille, 2018).

With the advantages of non-contact action, low residue and convenient application, microbial volatile fumigants have become a research hotspot in the field of fruit postharvest preservation. VOCs produced by streptomycetes have been verified to possess prominent antifungal and preservation effects (Khan et al., 2023; Tilocca et al., 2020; Almeida et al., 2023). Numerous studies have confirmed that streptomycetes can inhibit the growth of phytopathogens via releasing VOCs. Streptomycetes isolated from the cuticle of leafcutter ants can antagonize the pathogenic fungus *Escovopsis weberi* (Cuervo et al., 2023). VOCs from *Streptomyces sp*. SPS-33 and *Streptomyces setonii* WY228 can effectively control postharvest black spot of sweet potato, restrict lesion expansion and reduce fruit rot (Gong et al., 2022; Li et al., 2020).

In this research, the postharvest disease control and preservation efficacy of VOCs from TRM68247 were systematically verified at three levels: *in vitro* plate assay, detached twig test and wounded and pathogen-inoculated fruit experiment. Meanwhile, a horizontal comparison with previously reported biocontrol strains was conducted. In the plate assay, after 4 days of incubation, the mycelial growth inhibition rates of the VOCs against *V. pyri* and *A. gaisen* were 38.96% and 66.50%, respectively. Previous studies indicated that the antifungal activity of *S. rochei* A144 is highly susceptible to culture conditions, and its inhibition rate against *Valsa* mali ranges from 22.87% to 100% (Huang et al., 2024; Wang et al., 2023, 2025). *Bacillus atrophaeus* HF1 inhibits the mycelial growth of *V. pyri* with an inhibition rate of 61.20% (Yuan et al., 2023). Strain TRM68247 achieved effective antifungal activity after short-term incubation, suggesting that its postharvest preservation performance can be further improved by optimizing fermentation conditions.

For fungi of the genus *Alternaria*, the 66.50% inhibition rate of TRM68247 was slightly lower than that of *Bacillus velezensis* ZSY-1 (81.10%), but comparable to *Trichoderma asperellum* 2-X-3-3 (74.4%) and *S. rochei* ASH (63.75%–68.52%) (Yuan et al., 2022; Gao et al., 2017; Xu et al., 2026; Sudha et al., 2022). The results demonstrated that the disease control capacity of TRM68247 is equivalent to mainstream biocontrol microorganisms such a*s Bacillus* and *Trichoderma*.

In the detached pear twig fumigation test, the control efficacy of TRM68247 VOCs against pear valsa canker reached 74.91% after 14 days of treatment, which was slightly lower than that of the positive control eugenol (91.10%). Nevertheless, it was obviously superior to Bacillus amyloliquefaciens H12 with a field control efficacy of 55.56% and *B. atrophaeus* HF1. Hence, TRM68247 has practical application value in fumigation preservation and disease control for pear branches and postharvest fruits.

In the simulated postharvest storage assay of Korla fragrant pear, the control efficacies of TRM68247 VOCs against surface lesion expansion and internal flesh infection were 78.99% and 69.11%, respectively, which were significantly better than those of eugenol (Yuan et al., 2023; Sun et al., 2026). Different from *T. asperellum* 2-X-3-3 that alleviates postharvest black spot of pear merely through a single mode of action, VOCs from TRM68247 can not only suppress the spread of pathogens on fruit surfaces, but also block the invasion of pathogens into pulp tissues. It is speculated that these VOCs exert dual preservation mechanisms including direct antifungal activity and induced postharvest disease resistance of fragrant pear (Xu et al., 2026).

The differences in preservation efficacy between the two fumigants are mainly attributed to the distinct physicochemical properties of their active components. Microbial VOCs produced by TRM68247 have stable chemical properties, moderate volatilization rate and long duration of action. They can maintain effective antifungal concentrations throughout the whole storage period, inhibit mycelial growth and spore germination at the early stage of pathogen infection, and reduce fruit rot rate fundamentally. As a commonly used plant-derived antifungal agent, eugenol is highly volatile with rapid attenuation of active ingredients (Zhou et al., 2019). Its concentration decreases sharply in the late storage stage, easily leading to disease recurrence and poor preservation stability. Phenotypic observation and quantitative detection of pear fruits intuitively proved that fruits treated with TRM68247 VOCs had smaller lesions, milder pulp rot and better commercial appearance. By contrast, pathogen hyphae regrew and diseases continued to spread in the eugenol treatment group. In summary, VOCs from TRM68247 show stronger advantages in long-term antifungal activity, tissue penetration and delaying fruit senescence during postharvest preservation. In addition, a healthy fruit control fumigated with TRM68247 VOCs was not included, so potential phytotoxic effects on intact fruits cannot be fully ruled out.

Volatile organic compounds from actinomycetes are mainly synthesized via fatty acid metabolism, terpenoid biosynthesis and aromatic compound transformation pathways, and serve as key substances for non-contact fumigation preservation of postharvest fruits (Weisskopf et al., 2021). In this study, a total of 44 VOCs were identified from TRM68247 by gas chromatography-mass spectrometry (GC-MS). Five potential antifungal monomers were further screened out from eight dominant components. It should be noted that this selection may introduce potential bias into activity analysis; the screening was based on reported antifungal activity, commercial availability and cost of pure standards, and thus the bioactivity of the remaining three dominant VOCs was not evaluated in the present study. Among all detected compounds, 2-ethyl-1-hexanol exhibited the most prominent broad-spectrum antifungal and preservation activity, and this compound is widely found in soil microorganisms, endophytes and various streptomycetes (Ye et al., 2020; Yu et al., 2023; Wang et al., 2023). Activity assays showed that the minimum inhibitory concentration (MIC) of 2-ethyl-1-hexanol against the two postharvest pathogens of fragrant pear was both 5 × 10^−1^ μg⋅mL^(−1^, and its effective concentration was lower than most reported volatile antifungal compounds. Approved by the Joint FAO/WHO Expert Committee on Food Additives (JECFA) and the Flavor and Extract Manufacturers Association (FEMA), 2-ethyl-1-hexanol has low acute toxicity and favorable biodegradability, complying with the application standards for food and agricultural products (Joint Fao/Who Expert Committee on Food Additives., 2024). It is friendly to agroecosystems and human health, and thus is an ideal candidate for developing green fumigants for fruit postharvest preservation.

3,5-di-tert-butylphenol can damage the structure of fungal biofilms and reduce the pathogenicity of pathogens through dual regulatory pathways. It has strong specific inhibitory activity against *V. pyri* and moderate antifungal effects on *A. gaisen* at high concentrations (Rathna et al., 2016; Vijayakumar and MuhilVannan, 2021). The antifungal effect of nonanal is concentration-dependent. Previous studies have confirmed that nonanal can inhibit the growth of *Penicillium cyclopium* and *Botrytis cinerea* and induce host disease resistance. This study also verified its inhibitory activity against *A. gaisen* at high concentrations (Sun, 2017). In addition, although structural analogs of isomenthone and 2,3-dihydro-1H-inden-1-ol have been reported to possess potent antifungal potential, these two compounds showed no obvious activity in our in vitro validation (Sun et al., 2025; Jiang, 2025).

In conclusion, the extremophilic strain *S. rochei* TRM68247 represents a promising microbial resource for controlling postharvest fungal diseases and promoting green preservation of Korla fragrant pear. The abundant secondary-metabolite biosynthetic gene clusters (BGCs) and diverse volatile organic compounds (VOCs) produced by this strain indicate the feasibility of mining novel postharvest fumigants from extremophilic microorganisms. The dominant core components, 2-ethyl-1-hexanol and 3,5-di-tert-butylphenol, exhibited individual antifungal activity; further combination studies are warranted to clarify their interactions. With the advantages of ecological safety, low residue and non-contact fumigation, TRM68247 is well-suited for organic fruit preservation and postharvest storage of fragrant pear.

Future research should focus on two main directions: optimizing fermentation processes to improve the yield of core active VOCs and reduce the cost of industrial application, and further elucidating the molecular mechanisms underlying the antifungal activity and host resistance induction of VOCs, and provide theoretical support and high-value strain resources for the green and sustainable development of the fragrant pear postharvest industry.

## 5 Conclusion

*S. rochei* TRM68247, an extremophilic actinomycete isolated from rhizosphere soil of Taklamakan Desert, exhibits outstanding preservation and biocontrol capacity against two dominant fungal pathogens responsible for severe rot losses during the postharvest storage of Korla fragrant pear, *V. pyri* and *A. gaisen*. Genomic profiling uncovered that this strain harbors 47 secondary metabolite biosynthetic gene clusters together with abundant mobile genetic elements. These genetic endowments endow the strain with robust stress tolerance to harsh desert habitats and diverse metabolic capabilities, laying a firm genetic foundation for the biosynthesis of disease-suppressive volatile organic compounds (VOCs). GC-MS profiling successfully identified 44 volatile metabolites produced by TRM68247. Further in vitro bioassays validated 2-ethyl-1-hexanol and 3,5-di-tert-butylphenol as the core antifungal constituents. 2-ethyl-1-hexanol displayed low-dose, broad-spectrum suppression toward both pear pathogens, whereas 3,5-di-tert-butylphenol exerted potent targeted inhibition specifically against *V. pyri.* Consistently, fumigation trials on detached pear twigs and intact harvested fruits demonstrated that VOCs emitted by TRM68247 could sustainably retard lesion expansion and postharvest rot progression; several key control indices even outperformed eugenol, the widely adopted positive control plant-derived antifungal agent. Collectively, this work identifies a promising extremophilic *streptomycete* strain and its functional volatile components as potential resources for green postharvest disease management of fragrant pear. More importantly, our findings indicate that desert-originated extremophilic *actinomycetes* represent an underexplored reservoir for developing candidate microbial fumigants. The present study provides novel theoretical references and a feasible technical route toward sustainable postharvest preservation of fragrant pear fruit.

## Data Availability

The original contributions presented in this study are included in this article/Supplementary material, further inquiries can be directed to the corresponding authors.

## Footnotes

1
https://www.ezbiocloud.net/

2
https://www.ncbi.nlm.nih.gov

3
https://antismash.secondarymetabolites.org

4
https://card.mcmaster.ca/

5
https://imagej.net/
