# Supplementary material for: Volatile organic compounds from Streptomyces rochei TRM68247: fumigant biocontrol against postharvest diseases of Korla fragrant pear and identification of active antifungal components
Source: Front Microbiol. 2026 Oct 6;17:1930527. doi: 10.3389/fmicb.2026.1930527 (PMC13680479; doi:10.3389/fmicb.2026.1930527)
Supplement: Supplementary file 1 [file Table_1.docx]

**Figure S-1 Total ion current chromatogram (TIC) of TRM68247 by GC-MS**

**Supplementary material**

**Table S-1 Mobile genetic elements analysis in TRM68247 genome**

| Software | MGE_Type | Seqid | Start | End | Strand | Hit_Length | Contig_Length | Element_type |
| --- | --- | --- | --- | --- | --- | --- | --- | --- |
| ISEScan | IS | 2897 | 1 | 876 | + | 876 | 37250 | IS5 |
| ISEScan | IS | 2897 | 32828 | 33926 | - | 1099 | 37250 | IS5 |
| ISEScan | IS | 2899 | 2 | 877 | - | 876 | 39512 | IS5 |
| ISEScan | IS | 2977 | 83160 | 84501 | + | 1342 | 84503 | IS5 |
| ISEScan | IS | 2977 | 54663 | 58404 | + | 3742 | 84503 | ISNCY |
| ISEScan | IS | 2988 | 63736 | 65077 | - | 1342 | 65077 | IS5 |
| ISEScan | IS | 2999 | 404555 | 406363 | + | 1809 | 442431 | IS110 |
| ISEScan | IS | 3004 | 55090 | 56351 | - | 1262 | 85550 | IS256 |
| ISEScan | IS | 3004 | 1 | 876 | + | 876 | 85550 | IS5 |
| ISEScan | IS | 3004 | 1307 | 2386 | - | 1080 | 85550 | IS5 |
| ISEScan | IS | 3008 | 3 | 1344 | - | 1342 | 593902 | IS5 |
| ISEScan | IS | 3011 | 108694 | 110035 | - | 1342 | 110035 | IS5 |
| ISEScan | IS | 3019 | 668158 | 669883 | - | 1726 | 755235 | IS5 |
| ISEScan | IS | 3019 | 754358 | 755235 | + | 878 | 755235 | IS5 |
| ISEScan | IS | 3019 | 663047 | 664463 | + | 1417 | 755235 | IS701 |
| Platon | plasmid | 2908 | 1 | 11304 | . | 11304 | 11304 | . |
| Platon | plasmid | 2921 | 1 | 25515 | . | 25515 | 25515 | . |
| Platon | plasmid | 3010 | 1 | 6357 | . | 6357 | 6357 | . |
| Platon | plasmid | 3011 | 1 | 110035 | . | 110035 | 110035 | . |
| PhiSpy | phage | 3016 | 136916 | 183184 | . | 46269 | 407680 | . |
| ProphET | phage | 3016 | 154855 | 168570 | . | 13716 | 407680 | . |
| ProphET | phage | 3016 | 174094 | 206026 | . | 31933 | 407680 | . |

**Table S-2 Natural product biosynthetic gene clusters of the TRM68247 strains**

| Serial Number | Type | From | To | Most similar known cluster | | Similarity |
| --- | --- | --- | --- | --- | --- | --- |
| 1 | lassopeptide | 9,917 | 32,431 | [cattlecin](https://mibig.secondarymetabolites.org/go/BGC0001539/1) | RiPP | 75% |
| 2 | RiPP-like | 150,758 | 162,089 |  | | |
| 3 | NI-siderophore | 340,225 | 371,398 | [paulomycin](https://mibig.secondarymetabolites.org/go/BGC0001731/1) | Other | 13% |
| 4 | NRPS | 1 | 22,699 | [lipopeptide 8D1-1/](https://mibig.secondarymetabolites.org/go/BGC0001370/1)  [lipopeptide 8D1-2](https://mibig.secondarymetabolites.org/go/BGC0001370/1) | NRP | 45% |
| 5 | NRPS | 1 | 11,304 | [A54145](https://mibig.secondarymetabolites.org/go/BGC0000291/1) | NRP | 6% |
| 6 | T1PKS | 1 | 6,257 |  |  |  |
| 7 | T1PKS | 1 | 10,338 |  |  |  |
| 8 | NI-siderophore | 51,156 | 81,033 | [kinamycin](https://mibig.secondarymetabolites.org/go/BGC0000236/1) | Polyketide | 16% |
| 9 | T1PKS | 1 | 44,076 | [vicenistatin](https://mibig.secondarymetabolites.org/go/BGC0002634/1) | Polyketide+NRP+Other | 70% |
| 10 | hydrogen-cyanide | 51,516 | 61,116 | [aborycin](https://mibig.secondarymetabolites.org/go/BGC0002285/1) | RiPP | 28% |
| 11 | lanthipeptide-class-i | 131,115 | 155,712 |  | | |
| 12 | lanthipeptide-class-iii | 167,537 | 190,119 | [SapB](https://mibig.secondarymetabolites.org/go/BGC0000551/1) | RiPP:Lanthipeptide | 100% |
| 13 | T1PKS | 1 | 3,675 |  |  |  |
| 14 | NRPS | 1 | 7,621 | [coprisamide C/coprisamide D](https://mibig.secondarymetabolites.org/go/BGC0002378/1) | NRP | 8% |
| 15 | T1PKS | 1 | 10,722 | [mediomycin A](https://mibig.secondarymetabolites.org/go/BGC0001662/1) | Polyketide | 32% |
| 16 | terpene | 85,793 | 112,512 | [hopene](https://mibig.secondarymetabolites.org/go/BGC0000663/1) | Terpene | 100% |
| 17 | NI-siderophore | 176,172 | 205,944 | [desferrioxamin B/desferrioxamine E](https://mibig.secondarymetabolites.org/go/BGC0000940/1) | Other | 100% |
| 18 | melanin | 282,970 | 293,578 | [istamycin](https://mibig.secondarymetabolites.org/go/BGC0000700/1) | Saccharide | 4% |
| 19 | NRPS-like | 110,503 | 154,393 | [alanylclavam/](https://mibig.secondarymetabolites.org/go/BGC0000843/1)  [2-hydroxymethylclavam/](https://mibig.secondarymetabolites.org/go/BGC0000843/1)  [2-formyloxymethylclavam/clavam-2-carboxylate](https://mibig.secondarymetabolites.org/go/BGC0000843/1) | Other:Non-NRP beta-lactam | 12% |
| 20 | furan | 269,764 | 290,777 | [methylenomycin A](https://mibig.secondarymetabolites.org/go/BGC0000914/1) | Other | 9% |
| 21 | lanthipeptide-class-v | 296,498 | 338,830 | [pristinin A3](https://mibig.secondarymetabolites.org/go/BGC0002303/1) | RiPP | 11% |
| 22 | lanthipeptide-class-iii | 391,557 | 414,160 | [catenulipeptin](https://mibig.secondarymetabolites.org/go/BGC0000501/1) | RiPP:Lanthipeptide | 60% |
| 23 | T1PKS | 1 | 41,482 | [streptovaricin](https://mibig.secondarymetabolites.org/go/BGC0001785/1) | Polyketide | 26% |
| 24 | terpene | 169,257 | 190,288 |  | | |
| 25 | RiPP-like | 201,676 | 211,891 | [informatipeptin](https://mibig.secondarymetabolites.org/go/BGC0000518/1) | RiPP:Lanthipeptide | 42% |
| 26 | T1PKS | 1 | 6,357 | [salinomycin](https://mibig.secondarymetabolites.org/go/BGC0000144/1) | Polyketide  :Modular type I polyketide | 18% |
| 27 | NRPS | 181,946 | 217,749 | [CDA1b/CDA2a/CDA2b/CDA3a/CDA3b/CDA4a/CDA4b](https://mibig.secondarymetabolites.org/go/BGC0000315/1) | NRP:Lipopeptide:  Ca+-dependent lipopeptide | 47% |
| 28 | T2PKS,butyrolactone | 1 | 38,352 | [fluostatins M-Q](https://mibig.secondarymetabolites.org/go/BGC0001596/1) | Polyketide | 44% |
| 29 | terpene | 406,916 | 428,007 | [albaflavenone](https://mibig.secondarymetabolites.org/go/BGC0000660/1) | Terpene | 100% |
| 30 | T2PKS | 463,546 | 536,142 | [spore pigment](https://mibig.secondarymetabolites.org/go/BGC0000271/1) | Polyketide | 66% |
| 32 | T3PKS | 132,781 | 173,887 | [flaviolin/1,3,6,8-tetrahydroxynaphthalene](https://mibig.secondarymetabolites.org/go/BGC0002127/1) | Polyketide | 100% |
| 33 | NRPS-like | 416,431 | 458,419 | [streptothricin](https://mibig.secondarymetabolites.org/go/BGC0000432/1) | NRP | 100% |
| 34 | NRP-metallophore,NRPS | 10,793 | 69,166 | [coelichelin](https://mibig.secondarymetabolites.org/go/BGC0000325/1) | NRP | 100% |
| 35 | ectoine | 750,939 | 760,621 | [ectoine](https://mibig.secondarymetabolites.org/go/BGC0000853/1) | Other | 100% |
| 36 | terpene | 42,685 | 77,601 | [isorenieratene](https://mibig.secondarymetabolites.org/go/BGC0001227/1) | Terpene | 63% |
| 37 | indole | 135,465 | 156,586 | [5-dimethylallylindole-3-acetonitrile](https://mibig.secondarymetabolites.org/go/BGC0002128/1) | Other | 100% |
| 38 | terpene | 420,199 | 442,091 | [lysolipin I](https://mibig.secondarymetabolites.org/go/BGC0000242/1) | Polyketide | 4% |
| 39 | hglE-KS,T1PKS,NRPS-like,NRPS | 460,914 | 634,700 | [candicidin](https://mibig.secondarymetabolites.org/go/BGC0000034/1) | NRP+Polyketide | 95% |
| 40 | terpene | 696,990 | 722,573 | [isorenieratene](https://mibig.secondarymetabolites.org/go/BGC0000664/1) | Terpene | 100% |
| 41 | indole | 724,532 | 745,710 | [7-prenylisatin](https://mibig.secondarymetabolites.org/go/BGC0001294/1) | Other | 83% |

**Table S-3 Main Components Identified by Mass Spectrometry of VOCs of Strain TRM68247**

| **Peak No.** | **Retention Time** | **Peak Area** | **Peak Area %** | **Name** | **CAS No.** |
| --- | --- | --- | --- | --- | --- |
| 1 | 4.525 | 38173 | 0.30991 | Toluene | 108 - 88 - 3 |
| 2 | 5.615 | 2980259 | 24.19546 | Cyclotrisiloxane, hexamethyl- | 541 - 05 - 9 |
| 3 | 6.6 | 34386 | 0.27917 | Benzene, 1,3-dimethyl- | 108 - 38 - 3 |
| 4 | 7.499 | 1170185 | 9.50024 | 1H-Inden-1-ol, 2,3-dihydro- | 6351-10-6 |
| 5 | 8.524 | 8706 | 0.07068 | Octane, 2,6-dimethyl- | 2051 - 30 - 1 |
| 6 | 8.581 | 13752 | 0.11165 | Dimethyl trisulfide | 3658 - 80 - 8 |
| 7 | 8.885 | 78005 | 0.63329 | Heptane, 2,2,4,6,6-pentamethyl- | 13475 - 82 - 6 |
| 8 | 9.152 | 101448 | 0.82361 | Octanal | 124 - 13 - 0 |
| 9 | 9.622 | 288593 | 2.34296 | 1-Hexanol, 2-ethyl- | 104 - 76 - 7 |
| 10 | 9.714 | 31188 | 0.2532 | Butane, 1-[(1-methylethyl)thio]- | 7309 - 43 - 5 |
| 11 | 10.051 | 145399 | 2.1027 | Dodecane | 112 - 40 - 3 |
| 12 | 10.344 | 81252 | 0.65965 | 1-Octanol | 111 - 87 - 5 |
| 13 | 10.861 | 103098 | 0.83701 | 1,6-Octadien-3-ol, 3,7-dimethyl- | 78 - 70 - 6 |
| 14 | 10.861 | 784009 | 6.36504 | Nonanal | 124 - 19 - 6 |
| 15 | 10.933 | 433813 | 3.52194 | Benzaldehyde, 2,5-bis[(trimethylsilyl)oxy]- | 56114 - 69 - 3 |
| 16 | 11.86 | 18699 | 0.15181 | Tetradecane | 629 - 59 - 4 |
| 17 | 12.184 | 12170 | 0.0988 | 1-Dodecene | 112 - 41 - 4 |
| 18 | 12.381 | 21548 | 0.17494 | Methyl Salicylate | 119 - 36 - 8 |
| 19 | 12.436 | 12717 | 0.10324 | Decanal | 112 - 31 - 2 |
| 20 | 12.537 | 55560 | 0.45107 | Butane, 1-(propylthio)- | 1613 - 46 - 3 |
| 21 | 12.883 | 119433 | 0.96963 | Bicyclo[10.1.0]tridec-1-ene | 54766 - 91 - 5 |
| 22 | 13.214 | 10885 | 0.08837 | 1H-Cycloprop[e]azulene, decahydro-1,1,4,7-tetramethyl-, [1aR-(1a.alpha.,4.beta.,4a.beta.,7.beta.,7a.beta.,7b.alpha.)]- | 28580 - 43 - 0 |
| 23 | 13.279 | 70289 | 0.57065 | Santolina triene | 2153 - 66 - 4 |
| 24 | 13.437 | 43635 | 0.35425 | 3-Pentanol, 2,4-dimethyl- | 600 - 36 - 2 |
| 25 | 13.476 | 59155 | 0.48026 | n-Hexadecanoic acid | 57-10-3 |
| 26 | 13.523 | 27782 | 0.22555 | Nonanoic acid | 112 - 05 - 0 |
| 27 | 14.483 | 12218 | 0.09919 | 2-Decen-1-ol, (E)- | 18409 - 18 - 2 |
| 28 | 14.615 | 340741 | 2.76633 | Propanoic acid, 2-methyl-, 2-ethyl-3-hydroxyhexyl ester | 74367 - 31 - 0 |
| 29 | 14.71 | 24431 | 0.19834 | 1-Octanol, 2-butyl- | 735273 |
| 30 | 14.997 | 39946 | 0.3243 | 1-Pentadecene | 13360 - 61 - 7 |
| 31 | 15.213 | 14572 | 0.1183 | 2-Tridecanone | 593 - 08 - 8 |
| 32 | 15.417 | 2446435 | 19.86157 | Cyclohexanone, 5-methyl-2-(1-methylethyl)-, cis- | 491 - 07 - 6 |
| 33 | 15.77 | 52744 | 0.42821 | Heptanoic acid, propyl ester | 7778 - 87 - 2 |
| 34 | 15.837 | 66103 | 0.53666 | Linoleic acid ethyl ester | 544-35-4 |
| 35 | 15.929 | 38713 | 0.31429 | Copaene | 3856 - 25 - 5 |
| 36 | 16.094 | 24153 | 0.19609 | 2-Benzylcyclohexanone | 946 - 33 - 8 |
| 37 | 16.165 | 43518 | 0.3533 | 1H-Benzocyclohepten-7-ol, 2,3,4,4a,5,6,7,8-octahydro-1,1,4a,7-tetramethyl-, cis- | 552 - 02 - 3 |
| 38 | 16.363 | 67866 | 0.55098 | Hexadecane, 1-chloro- | 4860-03-1 |
| 39 | 16.606 | 2038590 | 16.55045 | Phenol, 3,5-bis(1,1-dimethylethyl)- | 1138 - 52 - 9 |
| 40 | 16.837 | 18491 | 0.15012 | .alpha.-Cubebene | 17699 - 14 - 8 |
| 41 | 18.442 | 17100 | 0.13883 | 2-Naphthalenemethanol, 1,2,3,4,4a,5,6,8a-octahydro-.alpha.,.alpha.,4a,8-tetramethyl-, [2R-(2.alpha.,4a.alpha.,8a.beta.)]- | 473 - 16 - 5 |
| 42 | 18.618 | 25108 | 0.20384 | 1-Nonadecene | 18435 - 45 - 5 |
| 43 | 18.748 | 122454 | 0.99415 | Cedranoxide, 8,14- | 18319 - 31 - 8 |
| 44 | 19.3 | 66509 | 0.53996 | 3-Buten-2-ol, 3-methyl-4-(2,6,6-trimethyl-2-cyclohexen-1-yl)- | 70172 - 00 - 8 |
